# Micromolding-based encapsulation of mesenchymal stromal cells in alginate for intraarticular injection in osteoarthritis

**DOI:** 10.1016/j.mtbio.2023.100581

**Published:** 2023-02-13

**Authors:** Fabien Nativel, Audrey Smith, Jeremy Boulestreau, Charles Lépine, Julie Baron, Melanie Marquis, Caroline Vignes, Yoan Le Guennec, Joelle Veziers, Julie Lesoeur, François Loll, Boris Halgand, Denis Renard, Jerome Abadie, Benoit Legoff, Frederic Blanchard, Olivier Gauthier, Claire Vinatier, Anne des Rieux, Jerome Guicheux, Catherine Le Visage

**Affiliations:** a Nantes Université, ONIRIS, Univ Angers, CHU Nantes, INSERM, Regenerative Medicine and Skeleton, RMeS, UMR 1229, F-44000 Nantes, France; b UCLouvain, Louvain Drug Research Institute, Advanced Drug Delivery and Biomaterials, 1200, Bruxelles, Belgium; c Nantes Université, CHU Nantes, Department of Pathology, F-44000 Nantes, France; d UR1268 BIA (Biopolymères Interactions Assemblages), INRAE, F-44300 Nantes, France; e LabONIRIS, ONIRIS (Nantes Atlantic College of Veterinary Medicine, Food Science and Engineering), F-44300 Nantes, France; f ONIRIS Nantes-Atlantic College of Veterinary Medicine, Centre de Recherche et D'investigation Préclinique (CRIP), F-44300 Nantes, France

**Keywords:** Rabbit, Hydrogel, Synovial fluid, Inflammation, Intra-articular, Cell therapy

## Abstract

Osteoarthritis (OA) is an inflammatory joint disease that affects cartilage, subchondral bone, and joint tissues. Undifferentiated Mesenchymal Stromal Cells are a promising therapeutic option for OA due to their ability to release anti-inflammatory, immuno-modulatory, and pro-regenerative factors. They can be embedded in hydrogels to prevent their tissue engraftment and subsequent differentiation. In this study, human adipose stromal cells are successfully encapsulated in alginate microgels via a micromolding method. Microencapsulated cells retain their in vitro metabolic activity and bioactivity and can sense and respond to inflammatory stimuli, including synovial fluids from OA patients. After intra-articular injection in a rabbit model of post-traumatic OA, a single dose of microencapsulated human cells exhibit properties matching those of non-encapsulated cells. At 6 and 12 weeks post-injection, we evidenced a tendency toward a decreased OA severity, an increased expression of aggrecan, and a reduced expression of aggrecanase-generated catabolic neoepitope. Thus, these findings establish the feasibility, safety, and efficacy of injecting cells encapsulated in microgels, opening the door to a long-term follow-up in canine OA patients.

## Graphical abstract

**Figure:**
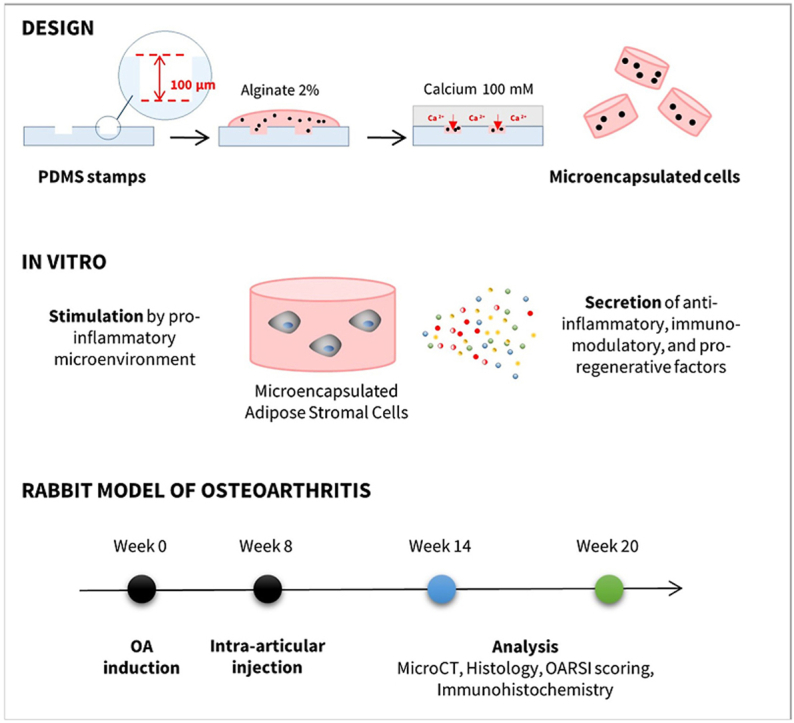

## 1 Introduction

Osteoarthritis (OA) is a multifaceted musculoskeletal disease that has become a major socio-economic problem worldwide [1]. According to the Global Burden of Disease Study, the disability-adjusted life years of OA patients increased by 105% from 1990 to 2016 [2]. A large proportion of this burden is due to hip and knee OA, with prevalent cases of 400 million worldwide [3,4]. First considered a disease mainly affecting the cartilage, it is now accepted that OA is characterized by several alterations appearing over time, including progressive cartilage damage, subchondral bone remodeling, osteophyte, and geode formation, and inflammation of the synovial membrane [5,6].

Existing therapeutic solutions provide symptomatic relief of pain at best but fail to prevent cartilage damage and destruction of other joint tissues [[7], [8], [9]]. In this context, mesenchymal stromal cells (MSCs)-based cell therapy has been increasingly considered a new approach to treating OA [10,11]. Initially isolated from bone marrow, these MSCs are now easily isolated from many other tissues, such as adipose tissue (ASC), umbilical cord, or synovial fluid, in clinically relevant quantities [12]. MSCs have been described for their protective effect on chondrocytes by reducing their apoptosis, hypertrophy, and dedifferentiation [13] and their anti-inflammatory properties. MSCs also exhibit immunoregulatory properties on T cell subsets and macrophages [14,15]. These properties partly rely on the abilities of MSCs to release trophic factors that mediate macrophage polarization (Prostaglandin E_2_ (PGE_2_), Tumor Necrosis Factor-stimulated gene 6 (TSG-6), Interleukin-6 (IL-6) and Indoleamine 2,3-dioxygenase (IDO)) and reduce chondrocyte hypertrophy and dedifferentiation (Hepatocyte Growth Factor (HGF) [13,16,17]. Consistently, the intra-articular (IA) injection of MSCs in OA animal models has been correlated with reduced disease progression, decreased pain, and inhibition of inflammation [[18], [19], [20], [21], [22]]. In humans, the IA injection of MSCs was also found to reduce pain and increase articular functionality [23,24]. Despite these encouraging results, the injection of MSCs in the joint space suffers two main limitations: (i) a massive cell death after injection into the joint space [25], (ii) and the risk of cell leakage outside the articular space [22,26].

Moreover, although MSCs were evidenced in different tissues of the joint (synovial membrane, articular cartilage) shortly after IA injection, the issue of their long-term persistence in an OA joint has been raised [27,28]. In immunocompetent mice, the low persistence of MSCs was confirmed, with less than 15% of cells found 10 days after injection in healthy and OA joints and no cells detected after 30 days [29]. Interestingly, several studies have undoubtedly demonstrated that multiple injections of MSC can improve and prolong the effects seen with a single injection, thereby suggesting that increasing the residence time of non-differentiated MSCs within the joint might increase their therapeutic effects [28,30,31]. To avoid the need for multiple injections, which are costly and risky for the patient, we thus hypothesized that the encapsulation of MSCs in a scaffold before a single IA injection would provide a physical anchor that would thus enhance their efficacy after a single IA injection [32].

Lin and collaborators recently encapsulated MSCs in a hyaluronic acid hydrogel modified with OA-targeted peptides designed to enhance joint lubrication [33]. After intra-articular injection in rat OA knee joints in a chemically induced rat model, they evidenced apparent cartilage regeneration at 8 weeks with enhanced Safranin-O staining and improved modified Mankin score, confirming encapsulation does not hinder the therapeutic effect MSCs. We also reported human ASCs (hASCs) encapsulation in alginate hydrogels [34]. Alginate is a natural linear polymer composed of blocks of (1,4)-linked β -d-mannuronate (M) and α -l-guluronate (G), with alternating M and G residues [35]. Due to its biocompatibility, biodegradability, low cost, and versatility, alginate has been extensively studied for pharmaceutical applications [35] and encapsulation of multiple cell types such as Langerhans islets, cardiac stem cells, and chondrocytes [36]. Alginate particles were generated by a dropwise extrusion of a cell-containing alginate solution through a calibrated needle into a crosslinking bath containing Ca^2+^ ions. We demonstrated that 1 ​mm diameter alginate particles supported hASCs ability to secrete therapeutic factors [34]. However, these large particles were not optimal for in vivo studies as they were too large to be injected into the knee of rodent models. A micromolding approach that enables the fabrication of calibrated micro-particles (less than 200 ​μm diameter) that are easily tunable by changing the shape and the size of the molds [37] was thus selected in this study.

In this context, the aims of this current study were (i) to demonstrate the feasibility of generating alginate-based microparticles using a cell-friendly micromolding technique, (ii) to assess the in vitro viability and bioactivity of hASCs encapsulated in these microparticles before and after injection and (iii) to evaluate the therapeutic efficacy of microencapsulated hASCs in a post-traumatic OA rabbit model.

## 2 Materials and methods

### 2.1 Materials

Polydimethylsiloxane (PDMS, RTV 615, used as a 2-part kit with a 10:1 mixing ratio) and SU-8 photoresist were obtained from Elecoproduit (France). Sodium alginate powder (Protanal™ LF10/60FT, 60–180 ​kDa, 25–35% mannuronic acid, and 65–75% guluronic acid) was purchased from FMC Biopolymer (USA). Phosphate-buffered saline (PBS) without calcium chloride and magnesium, Dulbecco's modified eagle medium (DMEM) high glucose (4.5 ​g/L), Hank's balanced sodium salt (HBSS), penicillin-streptomycin and trypsin/EDTA (0.05%/0.53 ​mM) were purchased from Invitrogen (Paisley, UK). Fetal calf serum (FCS) was obtained from Dominique Dutscher (Brumath, France). Calcium chloride (CaCl_2_) was purchased from VWR, and collagenase crude type I A, agarose, and citrate sodium from Sigma Aldrich. Synovial fluids were obtained from 9 patients with osteoarthritis (OA) and sampled during an arthrocentesis. Cells were removed by centrifugation before storage at −80 ​°C. The study was approved by the local ethics committee and the French Research Ministry (N°DC-2011-1399). All enrolled patients have given their formal consent. OA was diagnosed according to the EULAR criteria [38]. Patients with knee OA included 5 males and 4 females, with a mean age of 62 ​± ​8 (mean ​± ​SD). Synovial fluids were analyzed for interleukin 1 β (IL-1β), interleukin 6 (IL-6), interferon-gamma (IFN-γ), and tumor necrosis factor-alpha (TNF-α) using an ELISA kit (DuoSet®, R&D Systems, Canada), following the manufacturer's recommendations (Table 1).

**Table 1:** Synovial fluid analysis. Synovial fluid from OA patients (n ​= ​9) was analyzed by ELISA for tumor necrosis factor-alpha (TNF-α), interferon-gamma (IFN-γ), interleukin 1 β (IL-1β), and interleukin 6 (IL-6) content. KL: Kellgren Lawrence; N/A: not available. Patient inclusion criteria: adult patients (age >18 years); mechanical pain; knee joint effusion volume > 1 ​mL and abnormalities on radiological examination. Patient exclusion criterion: knee joint effusion volume < 1 ​mL.

Patient	Gender	Age	KL score	TNF-α (pg/mL)	IFN-γ (pg/mL)	IL-1β (pg/mL)	IL-6 (pg/mL)
A	Female	65	N/A	<3	<2.3	<7	1351
B	Male	64	IV	<3	<2.3	<7	174
C	Female	51	IV	3.9	4.1	<7	129
D	Female	58	0	36.6	<2.3	188	1180
E	Male	77	III	29.3	39.3	9214	264
F	Female	63	IV	<3	<2.3	<7	73
G	Male	58	0	<3	7.5	<7	2135
H	Male	54	IV	<3	<2.3	<7	25
I	Male	66	N/A	<3	<2.3	<7	431

### 2.2 Alginate micromolding

**Fig. 1:**
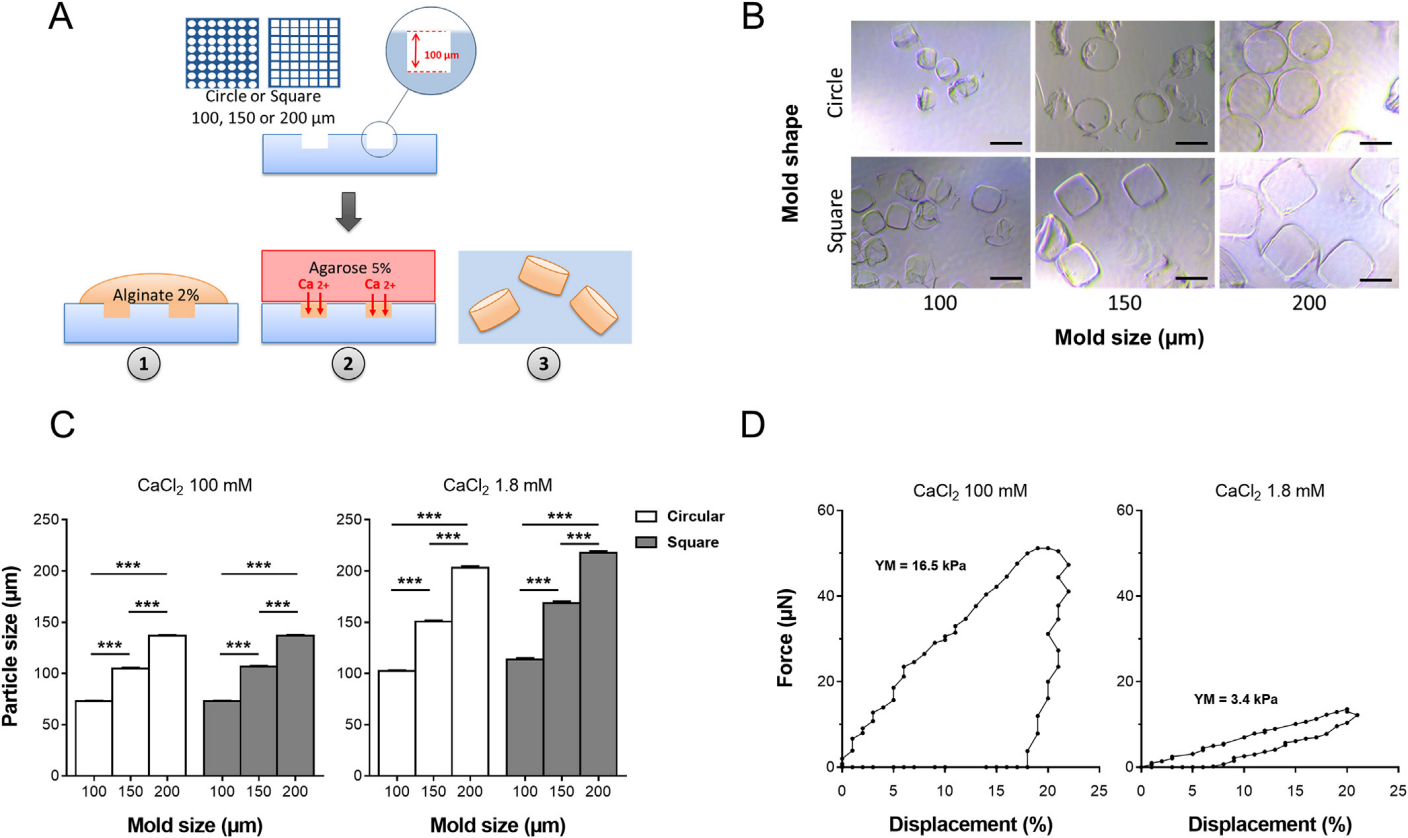
Morphological and mechanical characterization of micromolded alginate particles. (A) Microparticles were manufactured by pouring a sterile alginate solution (2% w/v) onto circular or square PDMS micromolds (100, 150, and 200 ​μm of diameter or side), followed by ionic cross-liking using an agarose gel loaded with 100 ​mM of CaCl2. (B) Microparticles were harvested and immediately observed under light microscopy. Scale bar 100 ​μm. (C) Alginate microparticles were stored in 100 ​mM CaCl2 or DMEM containing 1.8 ​mM CaCl2. After 24 ​h, the size of the microparticles was determined from digital images (n ​= ​30 particles imaged per condition). (D) Compressive properties of alginate microparticles (circular micromolds, 150 ​μm in diameter) stored in 100 or 1.8 ​mM of CaCl2 for 24 ​h were investigated by subjecting them to a 20% compression for 30 ​s. The force (μN) and displacement (%) were recorded, and Young's modulus (kPa) was calculated. Representative compression curves are shown. Results are expressed as means ​± ​SEM. ∗∗∗ represents a significant difference, p ​< ​0.001, two-way ANOVA, Tukey post-test. Abbreviations: PDMS: Polydimethylsiloxane; DMEM: Dulbecco's Modified Eagle's Medium.

Alginate micromolding was performed using polydimethylsiloxane (PDMS) micromolds by soft lithography. Briefly, PDMS was poured on a silicon wafer positively patterned by photolithography with SU-8 photoresist and cured overnight at 60 ​°C with an RTV615B catalyst kit, with a 10:1 mix rate ratio. Circular and square patterns of 100 ​μm in height, with 100, 150, and 200 ​μm diameter or side, respectively, were designed using the Adobe Illustrator software (Fig. 1A) to explore the influence of the pattern size and shape on micromolded particles properties. To enhance micromold handling, microchips containing clusters of 2 500, 1 600, and 900 patterns of 100, 150, and 200 ​μm size, respectively, were created. After curing, PDMS microchips were peeled from the silicon wafer. Microchips were hydrophilized using O_2_ plasma immediately before use (Zepto plasma system, Diener electronic; 40Watts, 90 ​s). For alginate microparticle generation, sodium alginate powder was sterilized by autoclave (134 ​°C, 4 ​min) and dissolved in sterile PBS (2% w/v). PDMS micromolds were filled by distributing 100 ​μL of the alginate solution on the top of the microchips, then scrapping the solution in excess with a cover slide. Alginate was crosslinked for 5 ​min using a calcium chloride-loaded agarose gel (5% w/v, CaCl_2_ 100 ​mM). After gelling, alginate microparticles were harvested from the microchips, transferred to Eppendorf tubes in a CaCl_2_ 100 ​mM solution or DMEM containing 1.8 ​mM of CaCl_2,_ and stored at 4 ​°C. The experiments were performed in triplicate for each condition.

### 2.3 Alginate microparticle characterization

The size and shape of microparticles were determined using a light microscope equipped with a digital camera (Nikon, Japan). Image analysis was performed using ImageJ 1.53 software. At least 30 particles were imaged for each condition.

Compressive mechanical properties were measured on alginate microparticles incubated in a 37 ​°C bath (CaCl_2_ 100 ​mM or 1.8 ​mM) using a Microsquisher® (CellScale, Waterloo, Canada). Each particle was compressed for 30 ​s with a square stainless-steel plate (1 ​mm) set on a 58 ​mm microbeam (152 ​μm diameter). The force (μN) and the displacement (μm) were recorded, and Young's modulus was calculated according to the manufacturer's recommendations.

E=stress/strain=(F/A)/(Δl/l0)

where E is Young's modulus; F is the force applied on a particle; A is the area onto which the pressure is applied; Δl is the displacement, and l0 is the initial diameter of the particle. The mechanical properties of at least nine microparticles were evaluated for each condition.

### 2.4 Alginate microparticles injectability tests

For these experiments, 1 ​mL syringes containing 5 ​× ​10^5^ microparticles of alginate (≈150 ​μm of diameter and 100 ​μm of height) in DMEM were connected to a 26G needle and settled in the texture analyzer TA.HDplus® (Texture Technologies, Hamilton, MA). Five hundred μL injections were performed using a 5 ​kg-load cell with a trigger force of 0.5 ​g and a speed test of 2 ​mm/s across a distance of 28 ​mm, corresponding to a speed of 2160 ​μL/min. The applied force (N) was recorded during 10s using Exponant software, and the results were exported as Excel files.

### 2.5 Adipose stromal cell isolation and cell culture

Human adipose-derived mesenchymal stromal cells (hASCs) were harvested as previously described [39]. Briefly, subcutaneous adipose tissue of 5 donors undergoing liposuction (Table S2), who had provided their informed consent, was collected, washed five times with Hank's balanced sodium salt (HBSS), then digested in collagenase (0.025% w/v in HBSS) for 1 ​h at 37 ​°C, under constant stirring. Complete culture medium (DMEM, 10% v/v fetal calf serum (FCS), 1% penicillin-streptomycin) was added to stop collagenase digestion, and the samples were centrifuged (300 ​g, 4min). The stromal fraction was collected, filtered through a 70 ​μm cell strainer, and centrifuged (300 ​g, 4 ​min). After supernatant removal, cells were suspended in a complete medium and seeded at 5000 ​cells per cm^2^. After 24 ​h, non-adherent cells were removed using PBS. Cells were then cultured in a complete medium at 37 ​°C (5% CO_2_, humidified atmosphere) and used in 2D and 3D experiments from passage 4 to passage 8. Micromolding set-up was first performed using cells from a single donor (Donor A). Subsequent in vitro experiments were performed with cells harvested from all five donors (Donors A-E, see Table S2). For in vivo evaluation, cells from two donors (Donors B and E) were used.

### 2.6 Cell encapsulation in alginate microparticles

First, cells were suspended in sterile 2% w/v alginate (3 million cells per mL). The cell suspension was then evenly distributed on the top of hydrophilised microchips. Cell loading was performed at room temperature by sedimentation (10 ​min) or centrifugation (300 ​g, 2 ​min). The excess suspension was removed, and alginate was crosslinked for 5 ​min using a calcium chloride-loaded agarose gel (5% w/v, CaCl_2_ 100 ​mM), as described above. After gelling, microparticles containing encapsulated cells were harvested from the microchips and transferred into Eppendorf tubes containing 1 ​mL of complete medium, with a total microchip corresponding to one sample transferred into one Eppendorf. Samples were cultured in a complete medium, at 37 ​°C (5% CO_2_, humidified atmosphere), with half of the medium changed every two days. In addition, microparticles were extruded through a 26G needle to mimic an injection into the joint, and their mechanical properties were determined as described above. In a separate experiment, the overall morphological and mechanical stability of micromolded alginate particles was analyzed overtime. The microparticles, with or without cells, were stored in DMEM containing 1.8 ​mM of CaCl_2_ at 37 ​°C. On days 14 and 28 after microfabrication, the microparticles were subjected to compression forces to 20% deformation, at a rate of 0.67 ​μm/s, using a Microsquisher® (CellScale, Waterloo, Canada). Height, diameter, and Young's modulus were evaluated for six microparticles for each condition.

### 2.7 Cell quantification and metabolic activity

Cell number and metabolic activity of encapsulated cells were determined 1 day and 7 days after encapsulation. The number of encapsulated cells was evaluated by DNA quantification using the CyQUANT® Cell Proliferation Assay (Invitrogen, UK), following the manufacturer's instructions. Briefly, 700 ​μL of the medium was removed from each sample and replaced by 700 ​μL of citrate sodium (60 ​mM in water) to reverse alginate gelation and disrupt the microparticle structure. After centrifugation (300 ​g, 4min), the supernatant was removed, the cell pellet was rinsed twice with PBS, and the nucleic acid stain reagent was added. The number of cells in each sample was then measured using a fluorescence microplate reader (ex 485 nm/em 530 ​nm) and normalized by the theoretical number of particles per sample to calculate the number of cells encapsulated in one single particle. In another experiment, 10% of PrestoBlue® Cell Viability Reagent (Invitrogen, UK) was added to the medium immediately or 6 days after encapsulation to evaluate cell metabolic activity. After 12 ​h of incubation, the supernatant fluorescence was measured using a fluorescence microplate reader (excitation 570 nm/emission 600 ​nm). Fluorescence was then normalized by the number of cells in the sample. In a separate experiment, cells were cultured for 2 months and their viability was assessed using a Live/Dead assay according to the manufacturer's recommendations and observed using confocal microscopy. Cells were also encapsulated using a fluorescent AlexaFluor 647-alginate (ratio alginate-Alexa647/alginate of 0.1%) synthesized through amidation of carboxylic acids with 4-(4,6-dimethoxy-1,3,5-triazin-2-yl)-4-methyl-morpholinium chloride. After 10 days of culture, the cells were analyzed with a Live/Dead assay and observed using confocal microscopy. In a separate experiment, microencapsulated cells were extruded and injected through a 26G needle to mimic an injection into the joint. Cell number and metabolic activity were then determined to evaluate the impact of injection on cell viability. For all subsequent experiments, the cells were encapsulated in alginate particles using circular micromolds with a 150 ​μm diameter, using the centrifugation technique. The experiments were performed six times for each condition, with cells from one donor (Donor A).

### 2.8 Cell secretory function

Microencapsulated cells were gradually deprived of fetal calf serum (10% on the day of encapsulation, 5% from day 1 to day 4, and 0.75% on day 4) to evaluate their ability to release cytokines and growth factors. Non-encapsulated cells were used as controls. On day 4, cells were stimulated with a culture medium containing 0.75% FCS, supplemented with 20 ​ng/mL of TNF-α (Miltenyi Biotec, Germany) and 20 ​ng/mL of IFN-γ (Miltenyi Biotec, Germany). Non-stimulated cells were cultured in a culture medium containing 0.75% of FCS. After 72 ​h (day 7 after encapsulation), the release of soluble factors into the supernatant was evaluated. Prostaglandin E_2_ (PGE_2_) concentration was determined using an ELISA kit (Cayman Chemical, USA), following the manufacturer's instructions. Human Growth Factor (HGF) and Transforming Growth Factor-beta (TGF-β) concentrations were also determined using an ELISA kit (DuoSet®, R&D Systems, Canada). Indoleamine 2,3-dioxygenase (IDO) enzymatic activity was measured through tryptophan-to-kynurenine conversion with a photometric determination of kynurenine concentration in the supernatant described before [34]. The experiments were performed in triplicate for each condition, with cells from four donors (Donors A-D, see Table S2). All results were normalized by the number of cells per sample.

Another experiment was performed using OA synovial fluids from 6 patients to stimulate the cells for 72 ​h. Encapsulated cells were gradually deprived of calf serum for 4 days and then stimulated with a culture medium containing 0.75% FCS supplemented with 10% v/v of synovial fluids harvested from OA patients (Patients A-F, see Table 1). After 72 ​h, PGE_2_ concentration and IDO activity were evaluated in the supernatant. The experiments were performed once for each condition, with cells from one donor (Donor A). All results were normalized by the number of cells per sample.

In another experiment, non-encapsulated and microencapsulated cells were mock injected through a 26G needle, gradually deprived of calf serum for 4 days, then stimulated with culture medium containing 0.75% FCS, supplemented with 20 ​ng/mL of TNF-α and 20 ​ng/mL of IFN-γ or supplemented with 10% v/v of synovial fluids harvested from 3 another OA patients (Patients G-I, see Table 1). Non-stimulated cells were cultured in a culture medium containing 0.75% of FCS. After 72 ​h, PGE_2_ concentration and IDO activity were evaluated in the supernatant. As a control, a study of the secretory function of cells in 2D monolayer culture was performed simultaneously. 2D experiments were performed using the same number of cells as 3D experiments, with 20 ​000 ​cells seeded in a 24-well plate. Cells were used at the same passage and were donor-matched to 3D experiments. The experiments were performed in triplicate for each condition, with hASCs from four donors (Donors B-E, see Table S2). All results were normalized by the number of cells per sample.

### 2.9 Animal experiments

Animal handling and surgical procedures were conducted at the Research and Preclinical Investigation Centre of the ONIRIS College of Veterinary Medicine, according to the European Community Guidelines for the care and use of laboratory animals (2010/63/UE) and approved by the national ethical committee (CEEA Pays de la Loire N°6, APAFIS#19415–2019 ​022 ​308 ​106 ​044 v3) and the institutional animal welfare committee at the ONIRIS College of Veterinary Medicine of Nantes.

### 2.10 Experimental OA model

We performed two separate animal experiments to assess the efficacy of encapsulated hASC. Twenty-four 15-week-old female New Zealand white rabbits were purchased from Charles River (Orleans, France). All animals had reached full skeletal maturity at the time of the study and weighed approximately 3.6±0.07 ​kg and 4.1±0.05 ​kg for the first and the second experiments, respectively. After one week of acclimatization, rabbits underwent a destabilization of the right joint induced by anterior cruciate ligament transection (ACLT) [40]. Eight weeks after surgery, animals were randomly assigned into 4 groups (n ​= ​6 animals per condition). They were injected through a 26G needle with 200 ​μL of either PBS, 25 ​000 blank microparticles in culture medium, 500 ​000 non-encapsulated cells in culture medium, or 500 ​000 ​cells encapsulated in 25 ​000 alginate microparticles. In this experiment, microchips with 10 ​000 micromolds were used to obtain a sufficient number of microencapsulated cells. Six or twelve weeks after injection, rabbits were euthanized by an overdose of barbiturates. All operated (right), and non-operated (left, contralateral sham: CL sham) joints were dissected and fixed in paraformaldehyde (4% w/v in PBS) for two days at 4 ​°C before imaging and histological analysis. The experiment was performed with cells from one human donor per animal experiment (Patients B and E).

### 2.11 Microcomputed tomography analysis

Joints were imaged using a Skyscan-1272 high-resolution 3D X-ray micro-computed tomography (micro-CT) system. The scanner was equipped with a 20–100 ​kV (10 ​W) X-ray source and an 11-megapixel X-ray detector. Each sample was placed on a holder with the sagittal suture oriented parallel to the X-ray detector and scanned using a 0.5 ​mm aluminum filter and 0.038 ​mm copper filter, 90 ​kV-11 μA, 26 ​μm isotropic voxels, 0.5°rotation step, and frame averaging of 3. For 3D reconstruction (NRecon 1.7.4.6® Skyscan, 2005–11®, Bruker microCT 2012–18) without smoothing, the ring artifact correction, beam hardening correction, and absorption coefficient were set to 3, 20%, and from 0.002 to 0.05, respectively. After image reconstruction, all datasets were automatically segmented for subsequent quantitative analysis of the joint thickness and tibial endplate, then subjected to sub-volume reconstruction for 3D volumetric analysis of bone erosion. First, cubic cuts (4.68 ​mm side – 180 cuts) were isolated on each side of the tibial endplate with Dataviewer software (1.5.6.2® 2004-11 Skyscan, 2012–17® Bruker microCT), then 2 regions of interest were isolated and cropped using a virtual punch (3.38 ​mm in diameter, 2.34 ​mm–90 cut height). After the pre-processing, structural bone parameters were calculated from the processed images using CTAn software (1.20.3.0® 2003-11 Skyscan, 2012–20® Bruker microCT). Six parameters were measured. Bone Volume to Tissue Volume ratio (BV/TV) represents the number of pixels classified as bone divided by the total number of pixels. The Subchondral Bone Plate Thickness (SBP.Th) and the rate of total porosity in the Subchondral Bone Plate (SBP. Po) were calculated. Regarding the trabecular bone, the Trabecular Thickness (Tb.Th), the Trabecular Separation (Tb.Sp), and the rate of total porosity in the Trabecular bone (Tb. Po) were calculated. The 3D Images were obtained using the CTVox Software (3.3.1® Bruker microCT).

### 2.12 Histological staining and OARSI scoring

After μCT imaging, joints were decalcified using EDTA 0.5 ​M (pH 7.4). After dehydration in a graded series of ethanol according to a predefined program (6 ​h in 80° ethanol, 6 ​h in 95° ethanol, 9 ​h in 100° ethanol, and then 9 ​h in methylcyclohexane), specimens were embedded in paraffin. Five levels and ten serial sagittal sections of 5 ​μm per level were cut using a microtome (RM 2255 Leica Biosystems, Nanterre, France). Safranin O/Fast green, Alcian blue, and Masson trichome stainings were then performed to visualize cells and extracellular matrix. The severity of OA lesions was scored according to a modified OsteoArthritis Research Society International (OARSI) scoring system (Table S3) [40] based on OARSI scoring [41,42]. Briefly, chondrocyte death, hypertrophy, cell clusters, loss of Safranin-O staining, surface alteration, and bone modifications were evaluated on one sagittal section per level, with three levels per joint. The mean scoring value was then calculated to obtain a score reflecting the severity of OA (maximum score 25). Three levels and one sagittal section were used to perform the OARSI scoring system. Three independent blind observers performed this histological assessment.

### 2.13 Immunohistochemistry and image analysis

Immunohistochemistry was performed on deparaffinized and rehydrated sections with primary antibodies directed against type II collagen (mouse antibody #CP18L, Calbiochem, France), antibodies directed against aggrecan (mouse antibody #MA3-16888, ThermoFisher, USA), and NITEGE (mouse antibody #MBS442004, My Biosource, USA) for the detection of aggrecan cleavage. First of all, antigen retrieval was carried out by incubation in either proteinase K (30 ​min 37 ​°C, 20 ​μg/mL #P6556, Sigma Aldrich, USA) or citrate buffer pH6 (10 ​min 100 ​°C or 3 ​h 70 ​°C) followed by incubation in hyaluronidase (15 ​min 37 ​°C, 1 ​mg/mL, #H3506, Sigma Aldrich, USA), for type II collagen and NITEGE immunostaining, respectively. For aggrecan immunostaining, antigen retrieved was carried out by chondroitinase (30 ​min at room temperature, 0.25U/mL, #C2905, Sigma Aldrich, USA) after reduction by dithiothreitol (2 ​h 37 ​°C, 10 ​mM, #D9760, Sigma Aldrich, USA) and alkylation by iodoacetamide (1 ​h 37 ​°C, 40 ​mM, #I1149, Sigma Aldrich, USA). Sections were then incubated with 3% (v/v) H_2_O_2_ (Sigma Aldrich, USA) to inactivate internal peroxidases. After blocking with 2.5% (v/v) horse serum (ref 30 ​022 #MP-7402 Vector Labs Burlingame, USA) for 30 ​min, sections were incubated overnight at 4 ​°C with the primary antibody solution (0.5 ​μg/mL for type II collagen, 10 ​μg/mL for *anti*-aggrecan, and 2 ​μg/mL for NITEGE in 0.1% (w/v) BSA). The sections were then incubated with peroxidase horse anti-mouse secondary antibodies (ref 30 ​028 #MP-7402, undiluted, Vector Labs) for 30 ​min at room temperature. The sections were developed with diaminobenzidine (DAB, #SK-4105, Vector Labs) for 3 ​min and counterstained using Mayer's hematoxylin (RAL Diagnostic, Martillac, France). Tissue sections were observed using Nanozoomer 2.0 Hamamatsu slide scanner (Hamamatsu Photonics, Hamamatsu, Japan) and imaged with NDP.view2 software® (Hamamatsu Photonics). Type II collagen, aggrecan, and NITEGE immunostaining were semi-quantified using QuPath® software [43] by measuring the diaminobenzidine *(*DAB) mean optical density (OD) in the articular cartilage matrix (20 measurements) and using the DAB mean OD in subchondral bone staining, as a blank (3 measurements). Then, the OD values were normalized to the mean intensity of cartilage in CL-sham joints. The results were expressed as an intensity ratio.

### 2.14 Statistical analysis

Graphpad 8® software was used for all statistical analyses. All results were reported as the mean ​± ​standard error of the mean, except for Micro-CT data and OARSI data that were presented as box-and-whisker plots with median, upper and lower values. A two-way unpaired ANOVA test followed by a Tukey post-test was used, with p values adjusted for multiple comparisons, to compare i) the size of alginate microparticles obtained from circular and square micromolds having 100, 150, and 200 ​μm of diameter or side; ii) the injectability of micromolded alginate particles with PBS or DMEM; iii) cell number and metabolic activity at day 1 and day 7 after encapsulation using sedimentation and centrifugation, iv) PGE_2_ concentration and IDO activity of encapsulated cells submitted to a mock injection or not injected, and v) the height, diameter and Young modulus of microparticles, loaded or not with cells, as a function of time. A two-tailed Mann-Whitney test was used to compare the number of cells encapsulated per particle using sedimentation and centrifugation. A Kruskal-Wallis test followed by Dunn's comparison test was used to compare the quantitative morphological parameters from microcomputed micrography analysis, the OARSI score, type II collagen, aggrecan, and NITEGE semi-quantification in rabbit joints.

## 3 Results

### 3.1 A cell-friendly micromolding protocol generates mechanically stable alginate particles

The microparticles were obtained by micromolding an alginate solution in circular or square PDMS micromolds of 100, 150, or 200 ​μm in diameter or side (Fig. 1A). After manufacturing and harvesting, the transparent microparticles were observed under light microscopy (Fig. 1B). Six types of particles were obtained, with well-defined cylindrical or rectangular geometries, depending on the shape of the micromolds, and almost no visible defect. Alginate particles were then stored in either a 100 ​mM CaCl_2_ solution or in a DMEM culture medium that contains a much lower physiological concentration of CaCl_2_ (1.8 ​mM) (Fig. 1C). When stored in a high concentration of CaCl_2_, the particles prepared with circular micromolds of 100, 150 and 200 ​μm in diameter measured 73.0 ​± ​0.6, 105.0 ​± ​0.7 and 136.8 ​± ​0.9 ​μm, respectively. When prepared with square micromolds of 100, 150, or 200 ​μm in side, the particles measured 72.9 ​± ​0.7, 107 ​± ​0.6, and 137.0 ​± ​0.8 ​μm, respectively. When the particles were transferred from 100 ​mM of CaCl_2_ to 1.8 ​mM of CaCl_2_, their dimensions significantly increased by about 40%, reaching a diameter of 102.5 ​± ​0.9, 150.8 ​± ​1.1, and 203.0 ​± ​1.8 ​μm for the 100, 150 and 200 ​μm in diameter circular micromolds, respectively, and a side of 113.7 ​± ​1.4, 168.7 ​± ​2.0 and 217.7 ​± ​1.7 ​μm for the 100, 150 and 200 ​μm in side square micromolds. Overall, the micromolding technique ensured the fabrication of microstructures with excellent resolution and fidelity, both from a size and shape point of view. For all subsequent experiments, the cells were encapsulated in alginate particles using circular micromolds with a 150 ​μm diameter. Compressive properties of alginate microparticles stored in 100 ​mM or 1.8 ​mM of CaCl_2_ were then investigated. The force required for a displacement of 20% was recorded when particles were subjected to a compression test for 30 ​s. Representative compression curves are presented in Fig. 1D. Displacement for a particle stored in 100 ​mM CaCl_2,_ and 1.8 ​mM CaCl_2_ showed a calculated Young's modulus of 16.5 ​± ​4.2 ​kPa and 3.4 ​± ​1.1 ​kPa, respectively. The alginate microparticles stored in 100 ​mM CaCl_2_ exhibited a significantly higher stiffness than those kept in a physiological concentration of CaCl_2_.

Microparticle injectability was also studied. Syringes containing the microparticles were connected to a 26G needle, and injection was performed in a texture analyzer TA.HD plus® and the applied force necessary to expel the microparticles was recorded during 10s. The result shows that the pressure applied to the syringe containing the microparticles is significantly increased compared to the syringe containing PBS or DMEM (0.90 ​± ​0.03 ​N vs. 0.56 ​± ​0,01 ​N and 0.51 ​± ​0.01 ​N, respectively) (Fig. S1). In any case, the required force remained well below the limit of injectability, set to 50 ​N to ISO 7886-1 standard.

### 3.2 Human adipose stromal cells survive after encapsulation in mechanically competent alginate microparticles

**Fig. 2:**
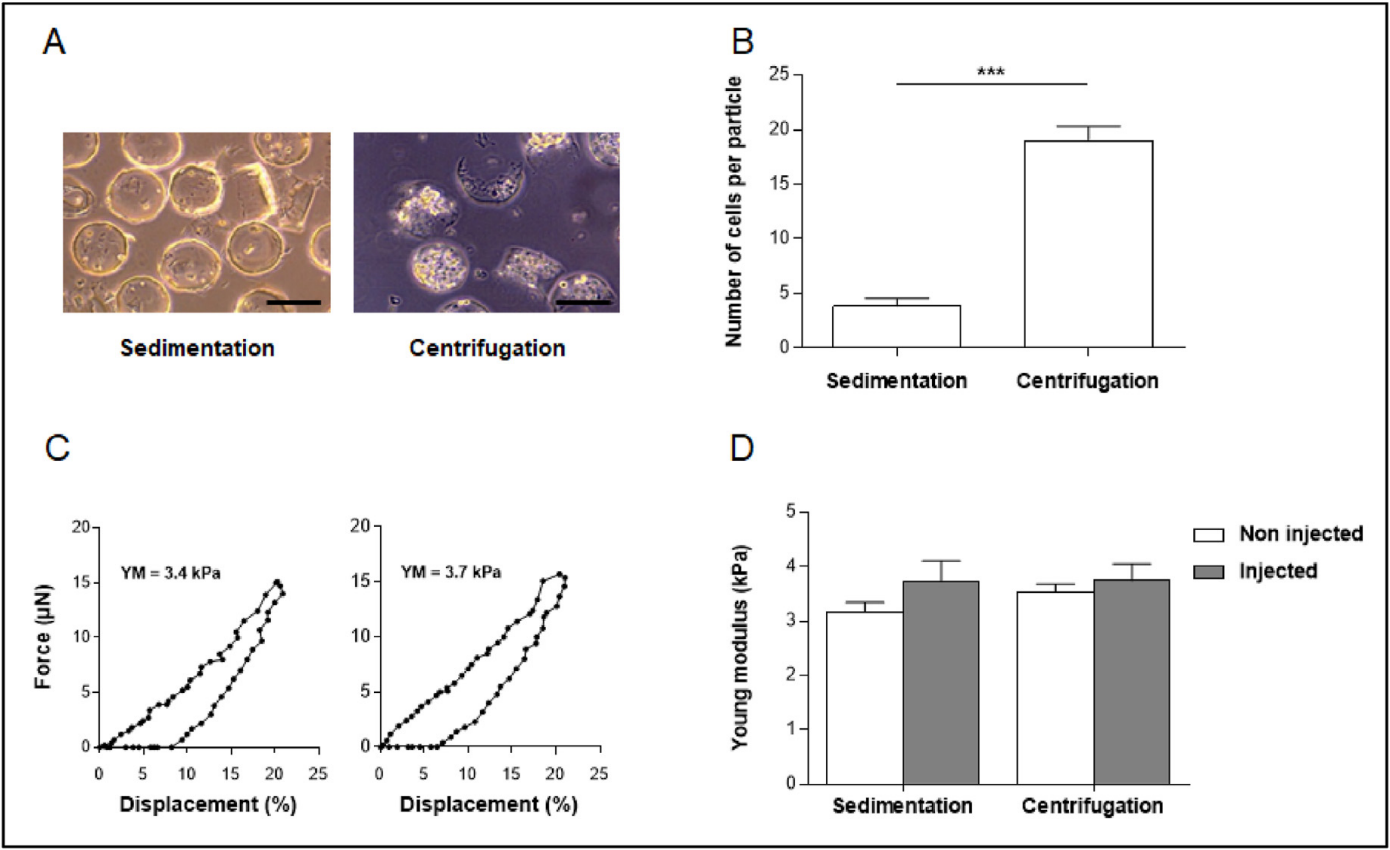
Cell encapsulation in micromolded alginate particles. Human adipose stromal cells were loaded into alginate microparticles prepared in circular micromolds with a 150 ​μm diameter. The cells were suspended in a sterile 2% (w/w) alginate solution and loaded into the molds by submitting the mold to sedimentation (1 ​g for 10 ​min) or centrifugation (300 ​g for 2 ​min). (A) After gelling, the particles were observed under light microscopy. Scale bar 100 ​μm. (B) The total number of encapsulated cells was determined by DNA quantification via CyQUANT assay and normalized by the theoretical number of particles to estimate the number of cells encapsulated per particle one day after encapsulation. Six independent experiments were performed with cells from donor A. (C) Compressive properties of alginate microparticles prepared using sedimentation (left) and centrifugation (right) techniques were investigated. The force (μN) and displacement (%) were recorded during compression (30 ​s), and the Young modulus (kPa) was calculated. (D) The Young modulus was also evaluated after a mock injection through a 26G needle (n ​= ​12). All results are expressed as means ​± ​SEM. ∗∗∗ represents a significant difference, p ​= ​0.022, Mann-Whitney test.

Human adipose stromal cells (hASC) were then encapsulated in round-shaped micromolds with a 150 ​μm diameter using the micromolding protocol (Fig. 2A), with a starting seeding density of 3 million cells per mL of alginate solution (2% w/v). Cell loading was performed at room temperature by sedimentation for 10 ​min before the cross-linking step with a calcium chloride-loaded agarose gel. Centrifuge-assisted micromolding was also performed to improve cell encapsulation, by submitting the micromolds to a centrifugation step at 300 ​g, for 2 ​min, before the cross-linking step. After crosslinking and harvesting, particles were transferred into a culture medium containing 1.8 ​mM of CaCl_2_ and observed using phase-contrast microscopy (Fig. 2A). Round-shaped particles presented an average diameter of 150.4 ​± ​1.5 ​μm, as assessed on digital images. Encapsulated cells were easily identified within the transparent alginate hydrogel, with a higher cell density observed for particles prepared with the centrifugation technique. The number of encapsulated cells per particle was assessed to confirm this observation (Fig. 2B). Using DNA quantification, we determined that the number of cells per particle was five times lower when the microparticles were prepared using the sedimentation technique compared to the centrifugation one (4 ​± ​1 and 19 ​± ​1 ​cells per particle, respectively). The mechanical properties of the particles loaded with cells were also studied. Representative displacement curves of particles under compression are presented in Fig. 2C. The average Young modulus of the particles prepared using sedimentation and centrifugation was similar (3.4 ​± ​1.1 ​kPa and 3.7 ​± ​0.7 ​kPa, respectively), indicating that the overall polymer network was not affected by the incorporation of cells during the cross-linking process (Fig. 2C). In addition, there was no significant difference with the Young modulus of particles prepared in the absence of cells and stored in 1.8 ​mM of CaCl_2_ (Fig. S2). Finally, the morphological and mechanical properties of the particles loaded with cells were evaluated after an injection through a 26G needle. The needle size was chosen based on previous injections into a rabbit knee. The particles' shape and size were similar before and after injection (data not shown). Furthermore, there was no significant difference in the Young modulus before and after injection (Fig. 2D), indicating that the alginate network was not modified after exposure to compressive forces during extrusion through a needle.

**Fig. 3:**
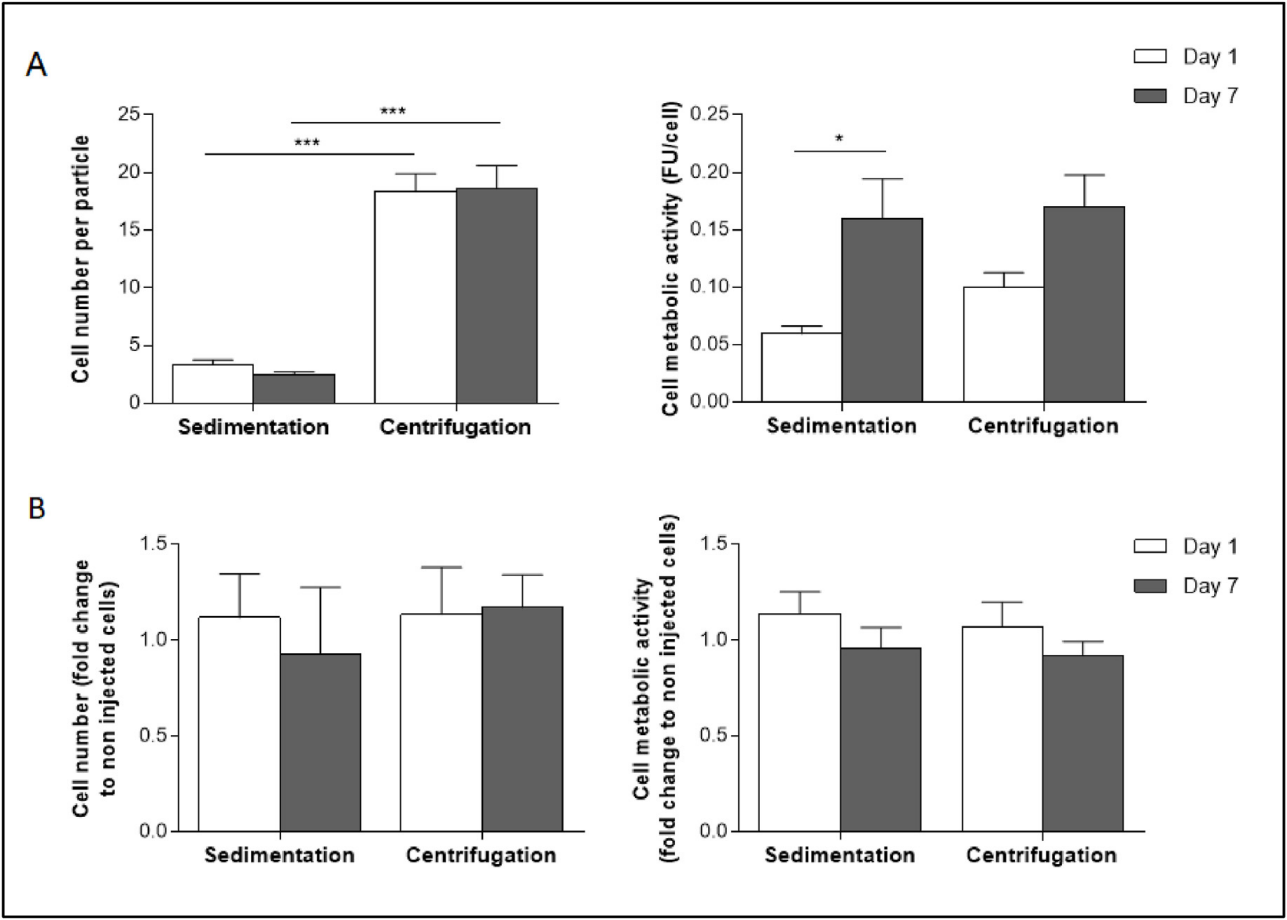
Cell number and metabolic activity after encapsulation. Human adipose stromal cells were encapsulated into alginate microparticles (prepared in circular micromolds with a 150 ​μm diameter) using a sedimentation (10 ​min) or a centrifugation (300 ​g, 2min) technique. (A) Cell number and cell metabolic activity (expressed in fluorescence unit) were evaluated on day 1 and day 7 after encapsulation via CyQUANT and PrestoBlue assays, respectively. The experiment was performed six times with cells from donor A. (B) In a separate experiment, encapsulated cells were extruded through a 26G needle to mimic an injection into the joint. Cell number and metabolic activity, determined via CyQUANT and PrestoBlue assays, respectively, were expressed as the fold change compared to non-extruded cells at each time point. Six independent experiments were performed with cells from donor A. The results are expressed as means ​± ​SEM. ∗ represents a significant difference, ∗p ​= ​0.039, ∗∗∗p ​< ​0.001, two-way ANOVA, Tukey post-test.

Alginate micromolded particles were then investigated for their ability to support cell culture. Cell viability was evaluated on day 1 and day 7 after encapsulation. The cell number per particle remained stable for 7 days after encapsulation, independently of the method used to load the cells into the alginate microparticles (Fig. 3A, left). Microparticles prepared by sedimentation contained 4 ​± ​1 ​cells on day 1 and 3 ​± ​1 ​cells on day 7, while microparticles prepared by centrifugation contained 18 ​± ​2 ​cells on day 1 and 19 ​± ​2 ​cells on day 7. From day 1 to day 7, the metabolic activity of cells loaded into the microparticles using sedimentation and centrifugation was multiplied by 3 and 2, respectively (Fig. 3A, right). Encapsulated cells were then submitted to injection through a 26G needle to mimic an injection into a rabbit joint. Cell number and metabolic activity were compared to those observed without injection (Fig. 3B). Results were expressed in fold change compared to the non-injected cells, a ratio of 1 indicating the absence of change in cell number and cell metabolic activity. Overall, injection through a 26G needle had no impact on cells' number and metabolic activity, whether they were microencapsulated by sedimentation or centrifugation. For cells microencapsulated by sedimentation, the cell number fold change was 1.12 ​± ​0.23 on day 1 and 0.93 ​± ​0.35 on day 7 (Fig. 3B, left), and the cell metabolic activity fold change was 1.13 ​± ​0.11 on day 1 and 0.96 ​± ​0.11 ​at day 7 (Fig. 3B, right), respectively. For cells microencapsulated by centrifugation, the cell number fold change was 1.14 ​± ​0.25 on day 1 and 1.17 ​± ​0.17 on day 7 (Fig. 3B, left), and the cell metabolic activity fold change was 1.07 ​± ​0.13 ​at day 1 and 0.91 ​± ​0.07 ​at day 7 (Fig. 3B, right), respectively. For all subsequent experiments, the cells were encapsulated in alginate particles using circular micromolds with a 150 ​μm diameter, using the centrifugation technique that enabled a significantly higher number of cells per particle. Morphological and mechanical stability of the alginate microparticles, loaded or not with cells, was evaluated in a culture medium at 37 ​°C. The alginate microparticles' height, diameter, and Young modulus remained stable on day 14 and day 28 (Fig. S2).

### 3.3 Human adipose stromal cells encapsulated in alginate microparticles synthesize Ido, PGE_2_, HGF, and TGF-β in response to TNF-α/IFN-γ or OA synovial fluids

**Fig. 4:**
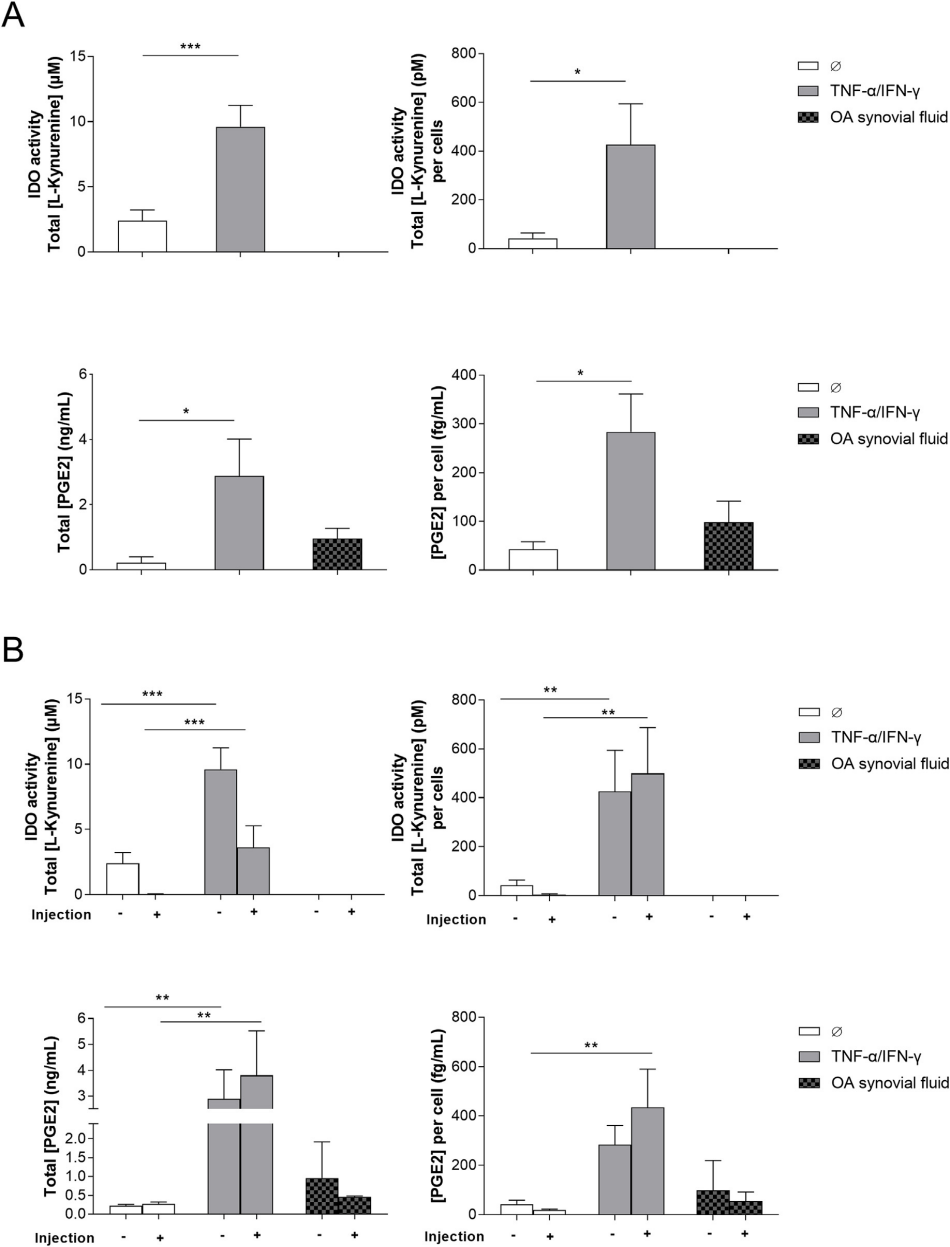
In vitro secretory function of microencapsulated cells. (A) Human adipose mesenchymal stromal cells were encapsulated in alginate microparticles (prepared in circular micromolds with a diameter of 150 ​μm) using centrifugation and then stimulated for 72 ​h with either TNF-α (20 ​ng/mL) and IFN-γ (20 ​ng/mL), or with synovial fluids from 6 osteoarthritic patients (dilution 1:10 in culture medium, unpooled). IDO activity and PGE_2_ concentration in the supernatants were evaluated. The results were normalized against the number of cells per sample and expressed as means ​± ​SEM. The experiment was performed in triplicate with cells from 4 donors (Donors A-D) for TNF-α/IFN-γ stimulation and OA synovial fluids from 6 patients (with cells from donor A). ∗ represents a significant difference, ∗p ​< ​0.05, ∗∗∗p ​< ​0.001, one-way ANOVA, Tukey post-test. (B) In a separate experiment, cells were encapsulated in alginate microparticles (prepared in circular micromolds with a diameter of 150 ​μm) and injected through a 26G needle, then stimulated for 72 ​h with TNF-α (20 ​ng/mL) and IFN-γ (20 ​ng/mL) or with synovial fluids from 9 osteoarthritic patients (dilution 1:10 in culture medium, unpooled). IDO activity and PGE_2_ concentration in the supernatants were evaluated. The results were normalized against the number of cells per sample and expressed as means ​± ​SEM. The experiment was performed in triplicate with cells from 5 donors (Donors A-E) for the ‘no injection’ condition and 3 donors (Donors B-D) for the ‘injection’ condition (with 2 donors (Patients B, D) used for IDO analysis) for TNF-α/IFN-γ stimulation. The experiment was performed in triplicate with cells from 3 healthy donors (A, D, and E) for the ‘no injection’ condition (n ​= ​9 OA synovial fluids) and cells from 2 healthy donors (D and E) for the ‘injection’ condition (n ​= ​3 OA synovial fluids). ∗ represents a significant difference, ∗∗p ​< ​0.01, ∗∗∗p ​< ​0.001, two-way ANOVA, Tukey post-test. Abbreviations. TNF-α: Tumor necrosis factor alpha; INF-γ: Interferon-gamma; IDO: Indoleamine 2,3-dioxygenase; PGE_2_: Prostaglandin-E2; OA: Osteoarthritis.

Encapsulated cells were then stimulated for 72 ​h with a pro-inflammatory stimulus, TNF-α (20 ​ng/mL), and IFN-γ (20 ​ng/mL) or with synovial fluids from 9 osteoarthritic patients. Indoleamine 2,3-dioxygenase (IDO) activity and prostaglandin E_2_ (PGE_2_) concentration in the supernatant were evaluated to assess the cell's ability to secrete anti-inflammatory and immunomodulatory factors (Fig. 4A). IDO activity and PGE_2_ concentration were also normalized to the total cell number per sample. After stimulation with TNF-α and IFN-γ, IDO activity was significantly increased, compared to unstimulated encapsulated cells (9.6 ​± ​1.7 ​μM vs. 2.4 ​± ​0.8 ​μM) (Fig. 4A, top left). After normalization by the cell number, IDO activity per cell was also significantly higher compared to unstimulated encapsulated cells (426.0 ​± ​168.3 pM vs. 42.5 ​± ​22.0 pM) (Fig. 4A, top right). Similarly, both PGE_2_ concentration (Fig. 4A, bottom left) and PGE_2_ concentration per cell (Fig. 4A, bottom right) were significantly higher compared to unstimulated encapsulated cells (2.9 ​± ​1.1 ​ng/mL vs. 0.2 ​± ​0.04 ​ng/mL and 283.5 ​± ​78.0 ​fg/mL vs. 42.7 ​± ​15.5 ​fg/mL, respectively).

In a separate experiment, cells were encapsulated, immediately injected through a 26G needle, then stimulated with TNF-α and IFN-γ for 72 ​h. IDO activity and PGE_2_ concentration in the supernatants were then determined (Fig. 4B) and normalized to the total cell number per sample. Whether the cells were injected or not, IDO activity was significantly increased, by a factor 98 and a factor 4, respectively, when the encapsulated cells were stimulated with TNF-α and IFN-γ, compared to unstimulated cells (Fig. 4B, top left). IDO activity per cell was also significantly increased upon stimulation with TNF-α and IFN-γ by a factor of 130 and 10 whether the encapsulated cells were injected or not, respectively (Fig. 4B, top right). Similarly, injection had no impact on PGE_2_ concentration (Fig. 4B, bottom left) and PGE_2_ concentration per cell (Fig. 4B, bottom right) when TNF-α and IFN-γ stimulated cells. Indeed, PGE_2_ concentration per cell was significantly increased upon stimulation whether cells were injected (435.5 ​± ​154.0 ​fg/mL vs. 19.7 ​± ​3.3 ​fg/mL for unstimulated cells), or not injected (283.5 ​± ​78.0 ​fg/mL vs. 42.7 ​± ​15.5 ​fg/mL for unstimulated cells). Similarly, the injection did not impact HGF and TGF-β concentrations per cell when cells were stimulated by TNF-α and IFN-γ (data not shown). Indeed, HGF concentration per cell was increased upon stimulation whether cells were injected (28.7 ​± ​15.9 ​fg/mL vs. 15.3 ​± ​9.2 ​fg/mL for unstimulated cells) or not injected (25.5 ​± ​11.3 ​fg/mL vs. 13.8 ​± ​5.0 ​fg/mL for unstimulated cells). TGF-β concentration per cell was increased upon stimulation whether cells were injected (23.5 ​± ​4.0 ​fg/mL vs. 14.3 ​± ​1.8 ​fg/mL for unstimulated cells) or not injected (16.9 ​± ​0.3 ​fg/mL vs. 14.5 ​± ​0.2 ​fg/mL for unstimulated cells).

To increase the clinical translatability of our data, we then sought to determine the effect of synovial fluids from 9 OA patients on the secretory activity of encapsulated cells. While IDO activity was not detected in the supernatant of encapsulated cells in the presence of pathological fluids (Fig. 4A and B, top row), both PGE_2_ concentration (Fig. 4A, bottom left) and PGE_2_ concentration per cell (Fig. 4A, bottom right) were increased by a factor 4 and a factor 2, respectively, compared to unstimulated cells. Similarly, injection had no impact on PGE_2_ concentrations (Fig. 4B, bottom left) and PGE_2_ concentrations per cell (Fig. 4B, bottom right) when cells were stimulated by synovial fluids harvested from OA patients. Indeed, PGE_2_ concentration per cell was increased upon stimulation whether cells were injected (54.5 ​± ​21.6 ​fg/mL vs. 19.7 ​± ​3.3 ​fg/mL for unstimulated cells) or not injected (98.5 ​± ​42.9 ​fg/mL vs. 42.7 ​± ​15.5 ​fg/mL for unstimulated cells).

At the same time, the secretory function of cells in 2D monolayer culture was analyzed as control of the 3D culture (i.e., microencapsulated cells). In 2D monolayer, IDO activity per cell significantly increased after stimulation with a pro-inflammatory stimulus (TNF-α and IFN-γ (20 ​ng/mL) or synovial fluids from OA patients) compared to encapsulated cells. Surprisingly, PGE_2_ concentration per cell significantly decreased in 2D monolayer compared to encapsulated cells (Table S1). In 2D monolayer culture, injection also had no impact on IDO activity and PGE_2_ concentration when cells were stimulated with pro-inflammatory stimulus.

### 3.4 Alginate-encapsulated hASCs prevent post-traumatic OA in a rabbit model

**Fig. 5:**
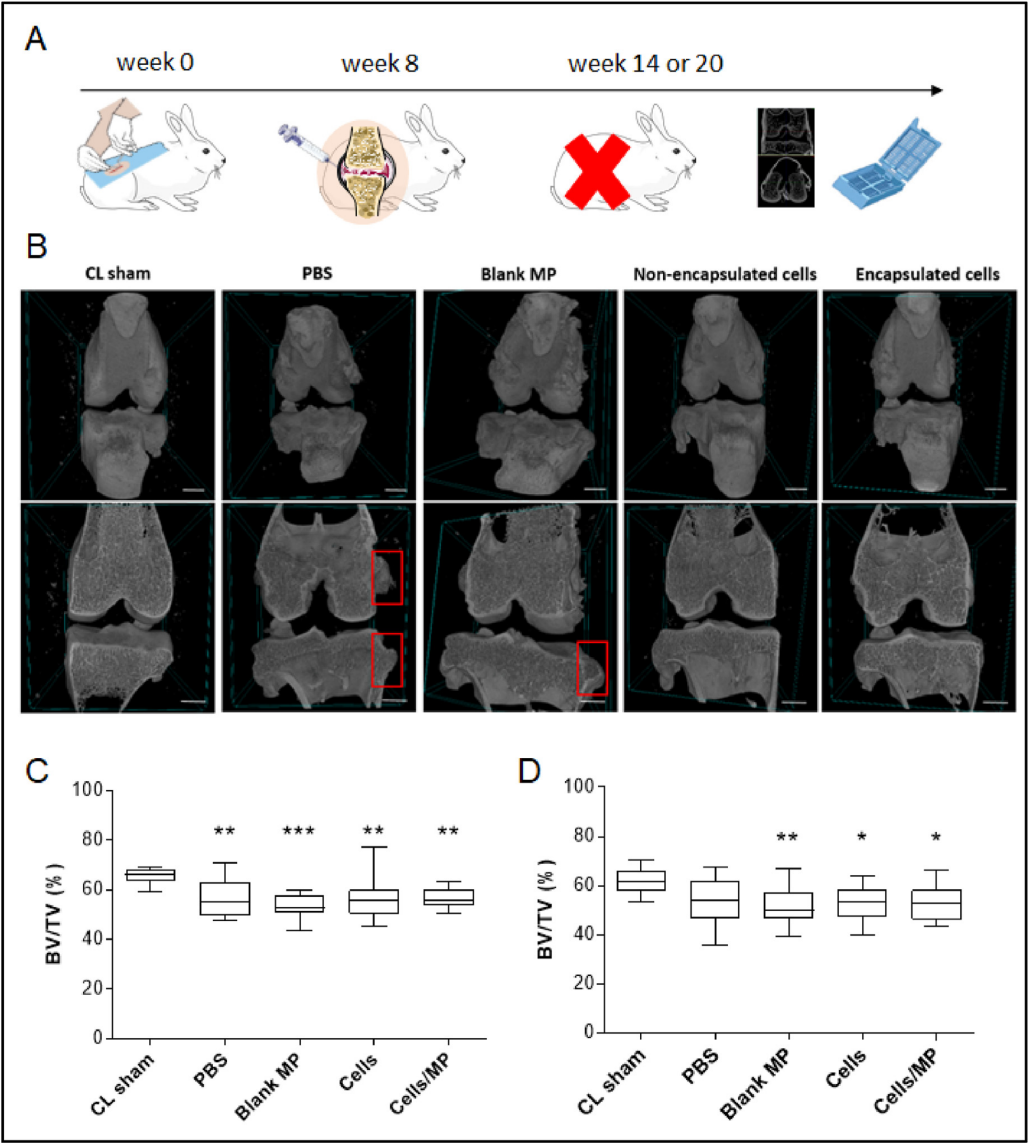
Validation of OA in a rabbit model. ACLT causes significant tibial epiphysis bone architecture changes after 14 or 20 weeks. Two independent animal experiments were performed to assess the time-course efficacy of encapsulated hASC. Rabbits (n ​= ​6 per condition) underwent a destabilization of the right joint induced by ACLT. (A) Eight weeks after surgery, animals were randomly assigned into 4 conditions. They were injected through a 26G needle with 200 ​μL of PBS, blank 2% (w/v) alginate microparticles (25 ​000 microparticles in 200 ​μL of culture medium, non-encapsulated human ASCs (500 ​000 ​cells in 200 ​μL of culture medium, or encapsulated human ASCs (500 ​000 ​cells in 25 ​000 2% (w/v) alginate microparticles in 200 ​μL of culture medium. Six or twelve weeks after IA injection, the rabbits were euthanized. All operated (right), and non-operated (left, contralateral sham: CL sham) joints were dissected and used for image analysis. The experiment was performed with cells from one human donor per animal experiment (Donors B and E). (B) Representative coronal micro-CT image of rabbit knee articulation (top: view from outside the joint, bottom: view from inside the joint) of all operated and non-operated joints at 20 weeks post-ACLT. Red frames indicate the presence of osteophytes. The experiment was performed with cells from one human donor (Donor E). Scale bar 0.5 ​cm. (C–D) Quantitative morphological assessment of bone tissue (BV/TV) of all operated and non-operated joints at 14 weeks (C) or 20 weeks (D) post-ACLT. ∗ represents a significant difference, ∗p ​< ​0.05, ∗∗p ​< ​0.01, ∗∗∗p ​< ​0.001, Kruskal-Wallis test followed by Dunn's comparison test, compared to CL sham. Abbreviations: OA: osteoarthritis, ACLT: anterior cruciate ligament transection, PBS: phosphate buffered saline, ASC: adipose-derived stromal cells, IA: intra-articular, MP: microparticles, BV/TV: bone volume to tissue volume ratio.

A post-traumatic osteoarthritis model was set up by performing an anterior cruciate ligament transection (ACLT) in adult rabbits (n ​= ​24, Fig. 5A) to assess the in vivo efficacy of microencapsulated hASCs over time. Two independent animal experiments were performed. Eight weeks after surgery, animals were injected with 200 ​μL of PBS, blank microparticles, non-encapsulated cells (500 ​000 ​cells), or encapsulated cells (500 ​000 ​cells). Rabbits were euthanized 6 or 12 weeks after injection to observe a time-course effect. The severity of OA in all operated (right) and non-operated (left, contralateral sham: CL sham) joints was assessed by coronal micro-computed tomography (micro CT) imaging. While CL sham joints exhibited no alteration during the study, all operated ACLT knees presented pathological changes, particularly in the ACLT animals injected with PBS or blank microparticles (Fig. 5B and Fig. S3). On average, 6 weeks after injection, the joint thickness of ACLT knees was significantly increased (2.6 ​± ​0.06 ​cm, n ​= ​24) compared to CL sham knees (2.0 ​± ​0.08 ​cm, n ​= ​6). Similarly, 12 weeks after injection, the joint thickness of ACLT knees was also significantly increased (2.5 ​± ​0.06 ​cm, n ​= ​24), in comparison with CL sham (1.9 ​± ​0.03 ​cm, n ​= ​6) (data not shown). Moreover, ACLT joints injected with PBS or blank microparticles exhibited an increased presence of osteophyte (highlighted with a red frame in Fig. 5B).

Micro-CT quantitative analysis was also performed on the tibial endplate. Overall, there was no significant difference between all experimental conditions, which were significantly different from the non-operated conditions. Indeed, we observed a significantly decreased Bone Volume/Tissue Volume ratio (BV/TV) in all ACLT joints compared to the CL sham joints (Fig. 5C and D). Six weeks after injection, ACLT joints exhibited a BV/TV ratio ranging from 53.4 ​± ​1.3% to 56.6 ​± ​1.1%, compared to a mean BV/TV ratio of 65.4 ​± ​0.9% for CL sham joints (Fig. 5C). This significant difference was also observed 12 weeks after injection. Indeed, ACLT joints exhibited a BV/TV ratio ranging from 51.3 ​± ​2.3% to 54.1 ​± ​2.3%, compared to a mean BV/TV ratio of 61.5 ​± ​1.5% for CL sham joints (Fig. 5D). Differences in the subchondral bone plate (SBP) of all operated animals were also observed (Figs. S4A–D). Six weeks after injection, the SBP thickness (SBP.Th) was slightly decreased in all operated joints, ranging from 343 ​± ​27 ​nm to 362 ​± ​47 ​nm, compared to the CL sham joint mean value of 401 ​± ​23 ​nm (Fig. S4A). Interestingly, 12 weeks after injection, all joints showed a lower SBP.Th than that measured on the joints in the first animal experiment. The SBP total Porosity (SBP.Po) was slightly increased in all operated joints, ranging from 85.0 ​± ​3.3% to 86.6 ​± ​2.0%, compared to CL sham joints mean value of 82.3 ​± ​1.4% for the first animal experiment (Fig. S4C) and ranging from 89.4 ​± ​1.5% to 90.7 ​± ​1.8%, compared to CL sham joints mean value of 85.7 ​± ​1.0% for the second animal experiment (Fig. S4D). Regarding the trabecular bone (Tb), its thickness (Tb.Th) was decreased in all operated joints, compared to the CL sham condition (258.8 ​± ​6.5 ​nm) in the first animal experiment (Fig. S4E). Moreover, the Tb.Th was significantly reduced in ACLT animals injected with blank microparticles (202.0 ​± ​5.2 ​nm) or non-encapsulated cells (218.8 ​± ​12.0 ​nm). In comparison, no significant reduction in Tb.Th was observed in the second experiment with Tb.Th ranging from 183 ​± ​10 ​nm to 200 ​± ​10 ​nm, compared to CL sham joint mean value of 214 ​± ​9 ​nm (Fig. S4F). Despite a reduction in the Tb Separation (Tb.Sp) between the 2 animal experiments, the Tb.Sp was similar in all operated and CL sham joints (Figs. S4G–H). Finally, in the first animal experiment, the trabecular bone total porosity (Tb.Po) was significantly increased in ACLT animals injected with blank microparticles (59.4 ​± ​1.6%) or non-encapsulated cells (58.5 ​± ​2.2%) compared to the CL sham condition (51.7 ​± ​1.2%) (Fig. S4I). In the same way, Tb.Po was significantly increased in ACLT animals injected with blank microparticles (56.5 ​± ​1.5%) or non-encapsulated cells (55.6 ​± ​1.4%) compared to the CL sham condition (50.1 ​± ​0.8%) (Fig. S4J).

**Fig. 6:**
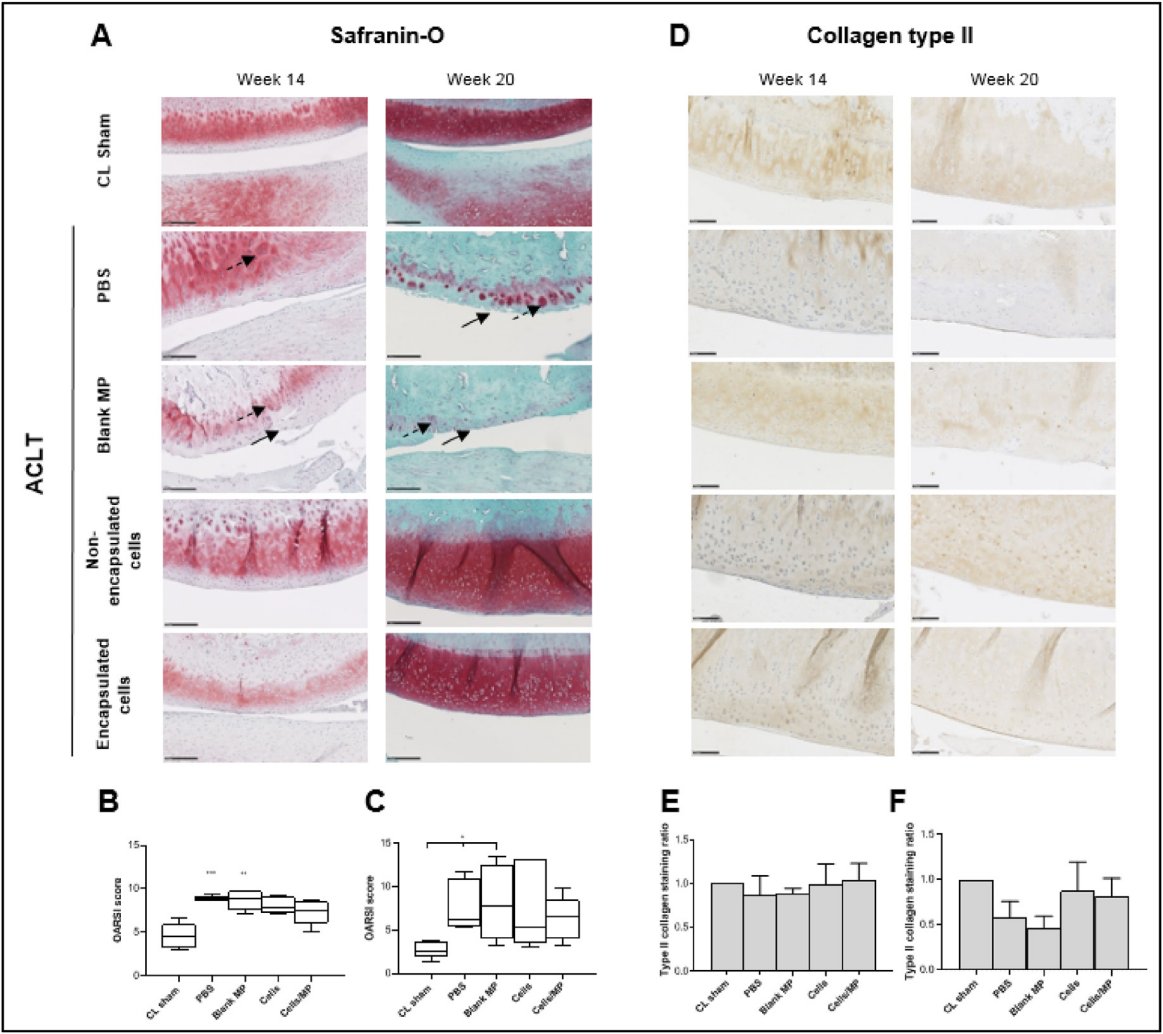
Histological analysis of OA in ACLT rabbit model. Two independent animal experiments were performed to assess the time-course efficacy of encapsulated hASC. Rabbits (n ​= ​6 per condition) underwent a destabilization of the right joint induced by anterior cruciate ligament transection (ACLT). Eight weeks after surgery, animals were randomly assigned into 4 conditions. They were injected through a 26G needle with 200 ​μL of PBS, blank 2% (w/v) alginate microparticles (25 ​000 microparticles in 200 ​μL of culture medium, non-encapsulated human ASCs (500 ​000 ​cells in 200 ​μL of culture medium, or encapsulated human ASCs (500 ​000 ​cells in 25 ​000 2% (w/v) alginate microparticles in 200 ​μL of culture medium. Six or twelve weeks after IA injection, the rabbits were euthanized. All operated (right), and non-operated (left, contralateral sham: CL sham) joints were dissected and used for histological analysis. The experiment was performed with cells from one human donor per animal experiment (Donors B and E). **(A)** Safranin-O staining of all operated and non-operated joints at 14 weeks or 20 weeks post-ACLT. Scale bar 250 ​μm. Black arrows indicate deep fibrillation or vertical fissures of the cartilage surface. Black dotted arrows indicate chondrocyte clusters in joint tissue. The severity of OA lesions was scored according to a modified OARSI score of all operated and non-operated joints at 14 weeks **(B)** or 20 weeks **(C)** post-ACLT. Briefly, chondrocyte death, hypertrophy, cell clusters, loss of Safranin-O staining, surface alteration, and bone modifications were evaluated. The mean scoring value was then calculated to obtain a score reflecting the severity of OA (maximum score 25). For each condition, three levels and one sagittal section were used (n ​= ​6 animals, except for the joint group injected by PBS in the 20-week study (n ​= ​5). The results are expressed as means ​± ​SEM. ∗ represents a significant difference, ∗p ​< ​0.05, ∗∗p ​< ​0.01, ∗∗∗p ​< ​0.001, Kruskal-Wallis test followed by Dunn's comparison test, compared to CL sham. **(D)** Representative immunohistochemical detection of type II collagen in operated and non-operated joints at 14 weeks or 20 weeks post-ACLT. Scale bar 100 ​μm. Type II collagen staining intensity in the cartilage matrix was semi-quantified at 14 weeks **(E)** or 20 weeks **(F)**, using subchondral bone staining as a negative control. Results were expressed as an intensity ratio. All measurements were performed using QuPath® software. Abbreviations: OA: osteoarthritis, ACLT: anterior cruciate ligament transection, PBS: phosphate buffered saline, ASC: adipose-derived stromal cells, IA: intra-articular, MP: microparticles.

After having demonstrated by micro-CT that a rupture of the rabbit ACLT significantly affected the subchondral bone architecture of the tibial epiphysis, we were interested in histologically determining whether joint tissues exhibit any cartilage extracellular matrix changes. Safranin-O staining was used to evidence the glycosaminoglycan content of the cartilage extracellular matrix. While CL sham joints showed no differences in articular cartilage (Fig. 6A and Fig. S6), all operated ACLT knees presented histological changes 14 or 20 weeks post-ACLT. Indeed, joints injected with PBS or blank microparticles revealed OA with deep fibrillations, vertical fissures, or delamination within the cartilage surface (Fig. 6A, black arrows), as well as a decreased number of chondrocytes in the superficial zone of the cartilage. In addition, some chondrocytes were organized in clusters in the superficial or the middle zone of the articular cartilage (Fig. 6A, black dotted arrows). In joints that were injected with non-encapsulated cells or encapsulated cells, the superficial zone of the articular cartilage was intact or presented a slight superficial abrasion. These histological observations were quantitatively confirmed using the modified Osteoarthritis Research Society International (OARSI) scoring. Six weeks after injection, all operated ACLT knees presented a significantly increased modified OARSI score as compared to CL sham, ranging from 7.3 ​± ​0.5 for encapsulated cells to 8.9 ​± ​0.1 for PBS, compared to 4.6 ​± ​0.6 for CL sham joints (Fig. 6B). To a greater extent, 12 weeks after injection, all operated ACLT knees presented a significantly increased modified OARSI score as compared to CL sham, ranging from 6.4 ​± ​1.0 for encapsulated cells to 8.1 ​± ​1.7 for blank microparticles, compared to 2.7 ​± ​0.4 for CL sham joints (Fig. 6C). For non-encapsulated cells and encapsulated cells, it is worth noting that the adjusted p values (values (ACLT conditions vs. CL Sham) were respectively 0.05 and 0.48 ​at 6 weeks post-injection and 0.06 and 0.08 ​at 12 weeks post-injection. On Alcian blue stained sections, slight discoloration on the joints' superficial zone of non-calcified cartilage injected with PBS or blank microparticles was observed (Fig. S5). In contrast, joints injected with non-encapsulated or encapsulated cells exhibited no difference from CL sham joints (black arrows). In the meniscal region of all ACLT joints, we also noted an alteration of the blue color, indicating a decreased glycosaminoglycan content (Fig. S5, black dotted arrows). Regarding the total collagen content of the articular tissue, Masson trichrome staining was similar in all operated and non-operated joints (data not shown).

**Fig. 7:**
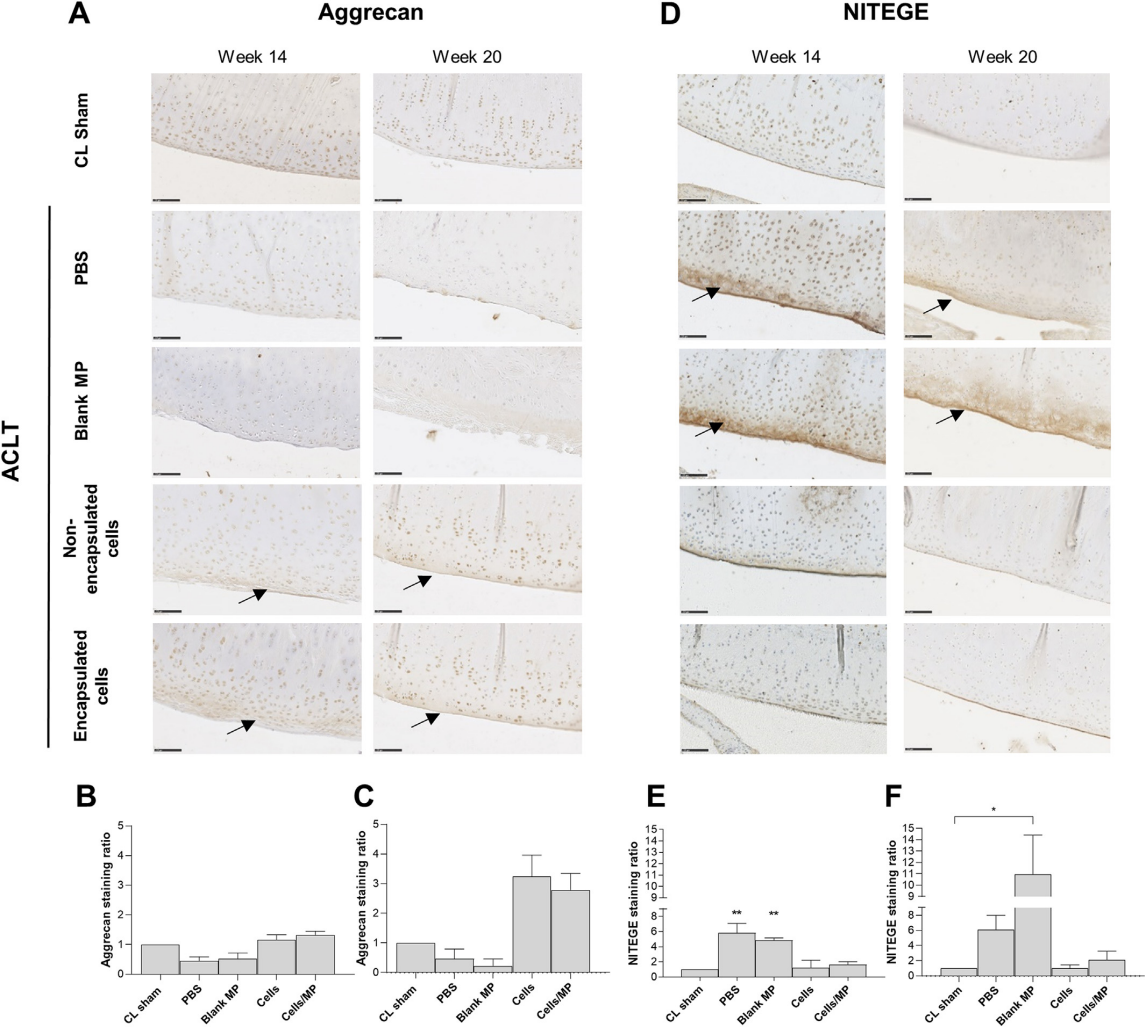
Histological analysis of ECM components in rabbit ACLT joints. Two independent animal experiments were performed to assess the time-course efficacy of encapsulated hASC. Rabbits (n ​= ​6 per condition) underwent a destabilization of the right joint induced by anterior cruciate ligament transection (ACLT). Eight weeks after surgery, animals were randomly assigned into 4 conditions. They were injected through a 26G needle with 200 ​μL of PBS, blank 2% (w/v) alginate microparticles (25 ​000 microparticles in 200 ​μL of culture medium, non-encapsulated human ASCs (500 ​000 ​cells in 200 ​μL of culture medium, or encapsulated human ASCs (500 ​000 ​cells in 25 ​000 2% (w/v) alginate microparticles in 200 ​μL of culture medium. Six or twelve weeks after IA injection, the rabbits were euthanized. All operated (right), and non-operated (left, contralateral sham ​= ​CL sham) joints were dissected and used for histological analysis. The experiment was performed with cells from one human donor per animal experiment (Donors B and E). (**A**) Representative immunohistochemical detection of aggrecan in operated and non-operated joints at 14 weeks or 20 weeks post-ACLT. Scale bar 100 ​μm. Black arrows indicate an intense pericellular aggrecan expression. Aggrecan staining intensity in the cartilage matrix was semi-quantified at 14 weeks (**B**) or 20 weeks (**C**) post-ACLT, using subchondral bone staining as a negative control. (**D**) Representative immunohistochemical detection of aggrecan epitope NITEGE in operated and non-operated joints at 14 weeks or 20 weeks post-ACLT. Scale bar 100 ​μm. Black arrows indicate an intense pericellular NITEGE expression. NITEGE staining intensity in the cartilage matrix was semi-quantified at 14 weeks (**E**) or 20 weeks (**F**) post-ACLT, using subchondral bone staining as a negative control. Results were expressed as an intensity ratio. All measurements were performed using QuPath® software. ∗ represents a significant difference, ∗p ​< ​0.05, ∗∗p ​< ​0.01, Kruskal-Wallis test followed by Dunn's comparison test, compared to CL sham. Abbreviations: OA: osteoarthritis, ACLT: anterior cruciate ligament transection, PBS: phosphate buffered saline, ASC: adipose-derived stromal cells, IA: intra-articular, MP: microparticles.

To further investigate whether encapsulated cells could prevent cartilage degradation after ACLT, immunohistochemical staining for type II collagen (Fig. 6D) and aggrecan (Fig. 7A) were performed. Type II collagen was observed throughout the joint sections, mainly as diffuse staining, with a slight decrease in the intensity for the joints that were injected with PBS or blank microparticles, compared to the CL sham condition and the joints that were injected with non-encapsulated cells or encapsulated cells. Semi-quantification showed no significant difference in type II collagen staining ratio in all joints associated with these slight differences (Fig. 6E and F). Aggrecan was predominantly observed in joint sections injected with non-encapsulated cells or encapsulated cells, as a diffuse intense staining, compared with CL sham conditions and joints injected with PBS and microparticles (Fig. 7A, black arrows). Semi-quantification of the aggrecan staining ratio revealed an increase of aggrecan content when the cells were injected non-encapsulated (1.2 ​± ​0.2 ​at 6 weeks after injection and 3.5 ​± ​0.7 ​at 12 weeks after injection) or encapsulated (1.3 ​± ​0.1 ​at 6 weeks post-injection and 2.8 ​± ​0.6 ​at 12 weeks post-injection) compared to CL sham condition (Fig. 7A and B).

Aggrecan degradation was evidenced by immunohistochemical detection of the C-terminal neoepitope NITEGE, exposed after aggrecanase cleavage (Fig. 7D). An intense pericellular NITEGE expression (black arrows) was observed in the non-calcified superficial cartilage of joints that were injected with PBS or blank microparticles, indicating a significant aggrecan degradation. Semi-quantification of the NITEGE staining ratio revealed a significant difference for ACLT joints that were injected with PBS or blank microparticles, in comparison with CL sham, with a fold increase of 5.8 and 4.9 respectively at 6 weeks post-injection (Figs. 7E), 6.1 and 11.0, respectively at 12 weeks after-injection (Fig. 7F). On the other hand, NITEGE staining ratio in the joints that were injected with non-encapsulated cells or encapsulated cells remained similar to that of CL sham joints, with a slight fold increase of 1.3 and 1.6, respectively at 6 weeks after injection and 1.0 and 2.0, respectively at 12 weeks after injection.

## 4 Discussion

In this study, we investigated the therapeutic potential of microencapsulated hASCs after intra-articular (IA) injection in a pre-clinical model of OA. ASCs have been shown to exert a protective effect on chondrocytes by reducing apoptosis and hypertrophy, as well as anti-inflammatory and immunomodulatory effects. Considering the poor survival rate of hASCs, several groups have explored cell encapsulation in hydrogels to anchor the cells within the articular space [33,44,45]. The hydrogel scaffold would provide a suitable 3D microenvironment supporting the biological activity of the hASCs, to decrease the severity and slow the progression of post-traumatic osteoarthritis. For hASC encapsulation, we selected sodium alginate, a natural polymer commonly used in cell therapy because of its biocompatibility and easy cross-linking [46,47]. Indeed, this versatile polymer undergoes a simple gelation process through ionic cross-linking with divalent cations such as Ca^2+^, with no need for chemical modification such as methacrylation under light UV exposure.

Alginate particle generation has been widely explored, and nowadays, a large panel of techniques exists to obtain calibrated microparticles as reviewed by *Lopes* et al. [48]: extrusion with the detachment of droplets by simple gravity [34,49], extrusion with forced droplet detachment by vibrational nozzle technology, or electrostatic bead generation [50,51], and emulsion/microfluidics technology [52,53]. The selection of the appropriate technique depends mainly on the targeted size and shape. We have considered the size of the rabbit articular space (800 ​μm - 1 ​mm) [54] and the internal diameter of a 26G needle (240 ​μm) to decide on our encapsulation technology. To our knowledge, this is the first report of the optimization of micromolding as a suitable method to obtain particles smaller than 200 ​μm, compatible with injection in a rabbit articular space.

Micromolding is a recently developed biofabrication technique of hydrogels with controlled size, shape, and topography. Hydrogel-forming polymers are cast into PDMS micromolds with predefined patterns generated by photolithography and subsequently gelled to form 3D constructs. Micromolding presents several advantages for cell encapsulation: contrary to emulsion technology, the use of a unique hydrophilic phase limits the effects of oils and surfactants that could impact cell viability or behavior, and there is no shear stress due to the cell suspension flowing through a nozzle. In addition, microparticles are easily demolded and harvested from the micromolds as long as the polymer does not interact with the PDMS microchip, and the hydrogel does not swell, which is the case with alginate.

Using round and squared micromolds, we successfully generated reproducible cylindrical and cubic alginate microparticles that were easily retrieved from the molds and cultured as a suspension. Although other studies have shown that alginate microparticle size is easily tunable [37,55,56], few studies have examined the impact of calcium concentration on the microparticle's final size, stiffness, and overall stability. As such, we worked with a physiological CaCl_2_ concentration (1.8 ​mM), which is 50 times lower than the prevalent cross-linking CaCl_2_ concentration (100 ​mM). As expected, the microparticle size was reduced in high CaCl_2_ concentration, with the formation of a denser network due to increased ionic interactions between divalent Ca^2+^ and negatively charged groups on the polymer chains [57]. The contraction of the alginate solution undergoing gelation in the micromolds facilitated the demolding and harvesting of the microparticles, whatever their cylindrical or cubic shape or size. Biomedical devices with sharp edges, such as rectangular or triangle-shaped ones, have been shown to induce a more intense foreign body reaction than those with a spherical shape [58]. Therefore, we have chosen to encapsulate cells using circular micromolds and selected a diameter of 150 ​μm compatible with an injection through a 26G needle (Fig. S1). This particle size is appropriate since the microgels will not be susceptible to rapid clearance via lymphatics and microvasculature [59]. Once transferred to a physiological calcium concentration, the microparticle size (150.8 ​± ​1.1 ​μm) was similar to the mold's original size, demonstrating the excellent size tunability and fidelity of the micromolding technique.

Regarding mechanical properties, a broad spectrum of Young Modulus values is reported in the literature for alginate hydrogels, ranging from 3 to 500 ​kPa [[60], [61], [62]]. This variability is explained by the lack of standardized methodology, with multiple molecular weights, polymer concentrations, methods of gelation (internal vs. external gelation), and incubation medium (culture medium, distilled water, calcium chloride bath) used. Surprisingly, most gelation protocols and measurements are performed in high CaCl_2_ concentrations (up to 5 ​M), hence not properly reflecting alginate hydrogels' behavior in physiological conditions. Since a standard range of human blood calcium concentration is 2.2–2.6 ​mM, and the synovial fluid is considered a plasma dialysate [63], we selected DMEM to mimic a physiological concentration of ∼2 ​mM and analyzed the microparticle elastic modulus and stability as a function of time. Measurements were performed with parallel-plate compression equipment in an aqueous bath at 37 ​°C. In line with the expected hydrogel contraction in high CaCl_2_ concentration, alginate microparticles stored in 100 ​mM CaCl_2_ exhibited a significantly higher stiffness than those at a physiological concentration. Incubation in a low CaCl_2_ concentration mimicking the articular conditions to anticipate the putative behavior of the particles in vivo decreased the elastic modulus by almost 5-folds, down to 3 ​kPa. These values are in agreement with recent studies reporting that the elastic modulus of 5% alginate microbeads could be tuned from 4 to 60 ​kPa when CaCl_2_ concentrations ranged from 10 to 100 ​mM [64].

A paramount criterion for ensuring the long-term efficacy of encapsulated cells is the stability of the alginate microparticles in the joint. Ionically crosslinked alginate hydrogels have previously shown poor stability in vitro, with an ion-exchange process with the local microenvironment leading to network disruption and hydrogel resorption. We thus investigated the microparticles from a morphological and mechanical point of view. Here, we observed that alginate microparticles remained physically stable for 28 days when incubated in vitro in physiological ionic conditions, with a constant diameter, height, and Young modulus (Fig. S2). Our results corroborate a previous study demonstrating that alginate hydrogels' long-term in vitro stability highly depends on calcium levels. While alginate beads stored in 0.9% w/v saline deteriorated in 2 weeks, those incubated in saline supplemented with a physiological concentration of calcium (2 ​mM) were intact after 3 weeks [57]. These results suggest that even if an ion-exchange process may still occur in the presence of physiological levels of calcium, alginate microparticles could persist within the joint cavity without swelling or disintegrating. Moreover, the persistence of alginate microbeads has been demonstrated in vivo for 4 weeks after subcutaneous implantation in mice and rats [[65], [66], [67]].

Cell encapsulation was performed by spreading a cell suspension over the micromolds. While cells settled into the mold via sedimentation and were observed in the particles after crosslinking, the cell density was low, around 3000 ​cells/mm^3^ of hydrogel, compared to 14 ​000 ​cells/mm^3^ for large alginate beads prepared by dripping [34]. As gravity alone was probably insufficient to drive the cells from the surface of the microchip into the molds, we performed a quick centrifugation step to force the cells into the molds. This step increased by 5 the number of cells encapsulated per particle, corresponding to a cell density of 12 ​000 ​cells/mm^3^ of the hydrogel. This centrifugation step had no apparent effect on cell survival and metabolic activity for 7 days after encapsulation compared to a standard dripping method [34] or to the sedimentation protocol. In a separate experiment, cells were labeled using a Live/Dead assay and observed with confocal microscopy after 2 months of culture (Fig. S7A). We also encapsulated the cells in fluorescent microparticles using alginate labeled with Alexa-647 fluorophore to localize the microparticle (Fig. S7B). The cells were randomly distributed, confirming that the centrifugation did not lead to the clumping of cells at the bottom of the microparticle. During the encapsulation, the cells are dispersed in a viscous alginate solution, and the centrifugation force applied to the cell culture plate is not strong enough to pellet the cells. During the culture period, we did not observe the formation of cell aggregates/spheroids, which could promote the maintenance of the stemness of the encapsulated hASC.

In addition to optimizing the encapsulation process, only this method provided a number of cells high enough to inject 500 ​000 ​cells/knee in a compatible volume. Indeed, with sedimentation, the required volume of microparticles would have been 170 ​μL, in a total injection volume of 200 ​μL, which leaves no room for the liquid vehicle. On the contrary, a much smaller volume (40 ​μL) of microparticles prepared by centrifugation was necessary. Hence the optimized centrifugation method was selected for the study.

In addition to cell survival, alginate microparticles should support cell biofunctionnality. We studied their ability to secrete proinflammatory factors PGE_2_ and IDO 10 after stimulation with TNF-α and IFN-γ to mimic the inflammatory environment found in OA joint. In agreement with a previous study using 1-mm diameter alginate beads [34], we demonstrated that cells encapsulated in 150-μm particles secreted PGE_2_ and IDO in response to a pro-inflammatory environment. It was slightly surprising to note that whatever the particles' size, the IDO's enzymatic activity and the concentration of PGE_2_, normalized to the number of encapsulated cells, were similar in both studies (around 500 pM/cell and 200 ​pg/mL/cell, respectively). Alginate hydrogel may thus provide an environment that allows the diffusion of molecules such as IDO (45 ​kDa) and PGE_2_ (352 ​Da) [68,69], in and out of the microparticle, independently of their sizes, up to 1 ​mm [34]. Indeed, in 1977, Andresen et al. indicated that the average pore size of the alginate network (i.e., porosity) was around 200 ​nm [70], which facilitates the in and out-diffusion of molecules less than 650 ​kDa [71]. In OA, an inflammatory environment persists; hence it would be relevant to know the secretory profile later than 3 days after stimulation. In this study, several experiments were conducted to assess the secretory profile of the cells at longer time points. However, cell death was evidenced after 7 days of culture in the pro-inflammatory medium that only contains 0.75% of serum. While the increased secretion of anti-inflammatory and immunomodulatory factors after stimulation by a pro-inflammatory environment was encouraging, it should be noted that we do not currently know exactly which of these factors are involved in OA and that all these in vitro conditions tested are far from reality in the human clinic. Indeed, OA synovial fluids include a large panel of molecules as previously reported [72] and as observed in our samples (Table 1). It is also important to highlight that the concentrations of TNF-α and IFN-γ used for in vitro stimulation are at least 1000 times higher than the ones in the synovial fluids harvested from OA patients. Based on those observations, we stimulated encapsulated cells with OA synovial fluids and evidenced an enhanced secretion of PGE_2_ compared to unstimulated cells. While cells were independently stimulated with synovial fluids from 9 patients (unpooled, diluted 10x in culture medium), the overall cell response was only half of the one obtained with TNF-α and IFN-γ. On the contrary, no IDO activity was detected after stimulation with OA synovial fluids. While the literature on human MSC stimulation with OA synovial fluids is scarce, one study reported a similar absence of IDO activity in vitro [73]. Upon stimulation of human MSCs by OA synovial fluids, IDO enzymatic activity was not detected in the supernatant, even though an increased expression of the genes encoding IDO was reported. In this study, the absence of IFN-γ in the OA synovial fluids could explain the lack of IDO activity [73]. Indeed, the link between IFN-γ and IDO gene expression has been demonstrated in multiple human cells, particularly in synovial cells [74], where the binding of IFN-γ to its receptors induces the expression of the gene encoding IDO. However, IDO gene expression is also induced by other signaling pathways independent of IFN-γ [75]. In our study, IFN-γ was evidenced in all OA synovial fluids, and cells were exposed to an average IFN-γ concentration of less than 1 ​pg/mL, using a 1:10 dilution. It is possible that our inability to detect the activity of IDO could be due not to its absence in the supernatant, but rather to a level of expression of the enzyme that was too low for its activity to be detected. A similar absence of IDO expression in the presence of OA synovial fluids was recently reported, even when hASCs were exposed to a 14 times higher concentration of IFN-γ [72].

As we aim at injecting the microencapsulated cells into a joint, the impact of injection on the cells was investigated. Although cell viability following injection is a genuine concern for stem cell-based therapy, the effect of injection remains unclear. While reduced viability of human MSCs has been initially described using a 30G [76] or a 28G needle [25], another study confirmed that cell viability, phenotype, and differentiation potential were not affected by injection through a 26G needle [77]. It is in agreement with our results, where a 26G needle did not induce any modification of the microencapsulated cell number and the cell metabolic activity and did not affect the alginate microparticle's morphological integrity. Moreover, we confirmed that stimulation by inflammatory cytokines induced the secretion of IDO, PGE_2_, HGF, and TGF-β by microencapsulated cells, whether cells were injected or not (Fig. 4B). To our knowledge, this is the first investigation demonstrating that injection through a 26G needle does not affect hASCs secretory activity.

To strengthen the translational relevance of our study, we then tested the injection of microencapsulated human ASCs in a rabbit model after destabilization of the right joint induced by anterior cruciate ligament transection (ACLT) [78,79]. ACLT induces a post-traumatic OA that takes longer to set up than chemically induced OA by intra-articular injection of enzymes (collagenases, papain) or molecules causing the death of articular chondrocytes (sodium mono-iodoacetate) [80]. This model is most representative of human traumatic and degenerative joint injuries. Among animal models, rabbit knees present the advantage of being easy to handle and are quite similar, at least in gross appearance, to those of humans [41,81]. We have chosen to work with mature female rabbits to avoid the known risk of spontaneous cartilage regeneration as much as possible, as previously reported [82]. Secondly, this model has the advantage of presenting an average articular space of around 1 ​mm [54], which is four times wider than those in rodents.

The effect of IA injection of MSCs on the progression of OA has been extensively studied in multiple animal models, mainly in rodents (mice and rats) [28,83] and in rabbits [[84], [85], [86]], with significant variability of the results, ranging from the ineffectiveness of the treatment to a decrease in the progression of the disease. The source and the types of cells used, the number of cells injected, the number of injections performed, and the nature of the injection medium are parameters that could influence the effectiveness of treatments [28,[83], [84], [85]]. In humans, the cell therapy efficacy depends on the number of cells, with a high-dose (100 ​× ​10^6^ ​cells) inducing clinical, radiological, and arthroscopic results that are more favorable than those in groups receiving a low (10 ​× ​10^6^ ​cells) or medium dose (50 ​× ​106 ​cells) [87]. Conversely, in another study, only the group of patients injected with a low-dose of hASCs (2 ​× ​106 ​cells) showed significant improvements in pain levels, and not those receiving medium (10 ​× ​106 ​cells) or high dose (50 ​× ​106 ​cells) [24]. Regarding rabbit pre-clinical data, several groups previously reported the IA injection of a cell suspension, either in culture medium [84], in a hyaluronic acid solution [85,88], or more recently in an innovative DNA supramolecular hydrogel [89]. Most studies used a medium dose between 1 ​× ​106 ​cells in 1 ​mL [84] or 400 ​μL [88], 2 ​× ​106 in 200 ​μL [89], or even a high dose (2.5 ​× ​106 ​cells) in 150 ​μL [85]. Using a similar ACLT model in rabbits, they reported a decreased progression of OA. The IA injection of alginate microencapsulated rabbit ASCs was recently shown to slow the progression of OA and reduce its extent. However, the number of injected cells was not reported, and the 3 injections were performed at 2, 3, and 4 weeks after surgery [86].

To our knowledge, our study is the first one describing the IA injection of micromolded alginate-encapsulated hASCs in a rabbit knee. In our study, a time course effect of injected hASCs was achieved by injecting a low dose of hASCs (i.e., 5 ​× ​10^5^ hASCs in 200 ​μL) six or twelve weeks after ACLT. Macroscopic and histological evaluation of disease progression was evaluated after euthanasia, 6 or 12 weeks post-injection, hence 14 or 20 weeks post ACLT. Using micro-CT, we confirmed that a rupture of the rabbit ACLT significantly affected the subchondral bone architecture of the tibial epiphysis as early as 6 weeks after surgery. Another group had already reported these observations 8 weeks post ACLT in rabbit model [90], with the growth of osteophytes [91]. In our study, in the lateral femoral condyle, where early cartilage degeneration has taken place, deep fibrillations or vertical fissures within the cartilage surface were observed, with a decreased number of chondrocytes organized in clusters in the superficial zone of the cartilage. The modified OARSI scoring has shown a tendency toward a reduced severity of OA lesions after injection of microencapsulated cells. In our study, the expression of type II collagen was similar in all experimental and control groups. Aggrecan expression has a tendency to increase over time in joints that were injected with non-encapsulated or encapsulated cells, suggesting a chondroprotective role exerted by injected hASC. This effect was probably due to HGF, a factor secreted by hASC known to increase proteoglycan synthesis in rabbit chondrocytes [92]. On the contrary, the detection of aggrecanase-generated catabolic neoepitope NITEGE indicated degenerative modifications in the rabbit joints (Fig. 7), consistent with the loss of GAGs evidenced on Safranin-O stained sections (Fig. 6). NITEGE is the prominent epitope associated with aggrecan depletion at an early time-point in various OA animal models [40] and OA patients [93]. As suggested by a previous report, injected ASC induced a decreased expression of proteases such as metalloproteinases (MMPs) and aggrecanases (ADAMTS-4), responsible for collagen and aggrecan degradation, respectively [94]. In this study, the reduced degradation of aggrecan suggests that a possible lesser expression of ADAMTS-4 might have slowed down the progression of cartilage damage.

Here, we were able to document that joints injected with a single low-dose of hASCs, whether encapsulated or not, exhibited up to 20 weeks post ACLT, a tendency toward a decreased OA severity compared to experimental groups injected with PBS or blank microparticles, thus confirming the therapeutic effect of encapsulated cells on the articular cartilage. Our findings established for the 1st time that the micromolding technology allows the manufacturing of microgels with a tunable size while maintaining the biological activity of the encapsulated cells, as well as the feasibility and safety of the delivery system. Further studies will require a long-term follow-up in canine OA patients.

In summary, we have generated micromolded alginate microparticles of 150 ​μm and demonstrated the long-term viability of microencapsulated hASCs and their ability to secrete factors such as PGE_2_, IDO, HGF, and TGFβ. We documented that joints injected with a single low-dose of hASCs, whether encapsulated or not, exhibited up to 20 weeks post ACLT, a tendency toward a decreased OA severity compared to experimental groups injected with PBS or blank microparticles, thus confirming the therapeutic effect of encapsulated cells on the articular cartilage. Our findings established for the 1st time that the micromolding technology allows the manufacturing of microgels with a tunable size while maintaining the biological activity of the encapsulated cells, as well as the feasibility and safety of the delivery system. Further studies are now warranted to investigate the therapeutic efficacy of microencapsulated cells and will require a long-term follow-up of the pain and functional gait in canine OA patients.

## Credit author statement

Conceptualization: JG, CLV, AdR, CV, DR. Investigation: FN, AS, JB, CL, JB, MM, CV, YLG, JV, JL, FL, BH, JA, BL, FB, OG. Supervision: CLV, AdR, JG. Writing – original draft: FN, AS, CLV, JG. Writing – review & editing: FN, AS, CLV, AdR, JG.

## Declaration of competing interest

The authors declare that they have no known competing financial interests or personal relationships that could have appeared to influence the work reported in this paper.

## Data Availability

Data will be made available on request.
