# Supplementary material for: Micromolding-based encapsulation of mesenchymal stromal cells in alginate for intraarticular injection in osteoarthritis
Source: Mater Today Bio. 2023 Feb 13;19:100581. doi: 10.1016/j.mtbio.2023.100581 (PMC9988569; doi:10.1016/j.mtbio.2023.100581)
Supplement: Multimedia component 1 [file mmc1.docx]

**Supplementary information**

**MESENCHYMAL STROMAL CELL ENCAPSULATION IN ALGINATE MICRO-PARTICLES FOR INTRA-ARTICULAR INJECTION IN OSTEOARTHRITIS**

Fabien Nativel ^1*^, Audrey Smith ^1,2*^, Jeremy Boulestreau ^1^, Charles Lépine ^3^, Julie Baron ^3^, Melanie Marquis ^4§^, Caroline Vignes ^1£^, Yoan Le Guennec ^1^, Joelle Veziers ^1^, Julie Lesoeur ^1^, François Loll ^1^, Boris Halgand ^1^, Denis Renard ^4^, Jerome Abadie ^5^, Benoit Legoff ^1^, Frederic Blanchard ^1^, Olivier Gauthier ^1,6^, Claire Vinatier ^1^, Anne des Rieux ^2#^, Jerome Guicheux ^1#^, and Catherine Le Visage ^1#^

* equivalent contribution

^#^ equivalent contribution

(1) Nantes Université, ONIRIS, Univ Angers, CHU Nantes, INSERM, Regenerative Medicine and Skeleton, RMeS, UMR 1229, F-44000 Nantes, France

(2) UCLouvain, Louvain Drug Research Institute, Advanced Drug Delivery and Biomaterials, 1200 Bruxelles, Belgium;

(3) Nantes Université, CHU Nantes, Department of Pathology, F-44000, France

(4) UR1268 BIA (Biopolymères Interactions Assemblages), INRAE, 44300 Nantes, France

(5) AMaROC, ONIRIS (Nantes Atlantic College of Veterinary Medicine, Food Science and Engineering), Nantes, France

(6) ONIRIS Nantes-Atlantic College of Veterinary Medicine, Centre de recherche et d’investigation préclinique (CRIP), F-44300, France

**Corresponding author**:

Catherine. Le Visage, ORCID ID: 0000-0003-1816-1649

Email: Catherine.levisage@inserm.fr

Nantes Université, ONIRIS, Univ Angers, CHU Nantes, INSERM, Regenerative Medicine and Skeleton, RMeS, UMR 1229, F-44000 Nantes, France

^§^ Current address: UMR0703 PAnTher (Physiopathologie Animale et BioThérapie du muscle et du système nerveux), INRAE, ONIRIS, 44307, Nantes, France

^£^ Current address: Nantes University, Univ Angers, INSERM, CNRS, Immunology and New Concepts in ImmunoTherapy, INCIT, UMR 1302/EMR6001. F-44000 Nantes, France

**Table S1. Secretary function of cells in 2D monolayer culture and condition (microencapsulation).** Abbreviations. TNF-α: Tumor necrosis factor alpha; INF-γ: Interferon-gamma; IDO: Indoleamine 2,3-dioxygenase; PGE_2_: Prostaglandin-E_2_; OA: osteoarthritis.

|  |  | IDO activity per cells (pM) | | [PGE2] per cells (fg/mL) | |
| --- | --- | --- | --- | --- | --- |
| Stimulation by |  | 2D | 3D | 2D | 3D |
| TNF-α/ IFN-γ | No injection | 1310±284 | 426.0±168.3 | 1.8± 0.3 | 283.5±78.0 |
|  | Injection | 1301±273 | 499.5±187.7 | 1.5±0.2 | 435.5±154.0 |
| Synovial fluids  from OA patients | No injection | 0 | 0 | 4.6±0.7 | 98.5±42.8 |
|  | Injection | 0 | 0 | 4.1±0.7 | 54.5±21.6 |

**Table S2. Donors of human adipose-derived mesenchymal stromal cells**

| Donor | Gender | Age (years) | Experiments |
| --- | --- | --- | --- |
| A | Female | 60 | Micromolding set-up, in vitro encapsulation, and stimulation |
| B | Female | 52 | In vitro encapsulation and stimulation, in vivo injection |
| C | Female | 46 | In vitro encapsulation and stimulation |
| D | Man | 40 | In vitro encapsulation and stimulation |
| E | Female | 56 | In vitro encapsulation and stimulation, in vivo injection |

**Table S3. Modified OARSI score**

| Chondrocytes death | None | 0 |
| --- | --- | --- |
|  | ≤ 10% | 1 |
|  | ≤ 25% | 2 |
|  | ≤ 50% | 3 |
|  | ≤ 75% | 4 |
|  | ≤ 100% | 5 |
| Hypertrophy | None | 0 |
|  | Presence | 1 |
| Clusters | None | 0 |
|  | Superficial zone | 1 |
|  | Middle zone | 2 |
|  | Complex structures | 3 |
|  | Mild layer cyst formation | 4 |
| Loss of Safranin-O staining | None | 0 |
|  | Superficial zone of non-calcified cartilage | 1 |
|  | Middle zone of non-calcified cartilage | 2 |
|  | Non-calcified cartilage and intense staining around clusters | 3 |
|  | Absent with staining around clusters | 4 |
|  | Absent | 5 |
| Surface | Intact | 0 |
|  | Superficial fibrillation or abrasion | 1 |
|  | Deep fibrillation | 2 |
|  | Vertical fissures | 3 |
|  | Delamination/ excavation | 4 |
| Bone | None | 0 |
|  | Denudation | 4 |
|  | Microfracture | 5 |
|  | Remodeling | 6 |
|  | Total | 25 |

**Figure S1. Injectability test of micromolded alginate particles without encapsulated cells.** Alginate microparticles were prepared in circular micromolds with a 150 µm diameter. 2% (w/v) alginate solution was charged into the molds by submitting the mold to centrifugation (300g for 2 minutes). After ionic cross-liking using an agarose gel loaded with 100 mM of CaCl_2_, alginate microparticles were stored in DMEM containing 1.8 mM CaCl_2_ at 37 °C. 1 mL syringes containing 5 x 10^5^ microparticles in DMEM were connected to a 26G needle and settled in the texture analyzer TA.HDplus^®^. Five hundred μL injections were performed. The applied force was recorded during 10s (n=3 for each condition). Results are expressed as means ± SEM. The result shows that the force applied to the syringe containing the microparticles is significantly increased compared to the syringe containing PBS or DMEM, p<0.0001, two-way ANOVA, Tukey post-test. Abbreviations: PBS: phosphate buffered saline; DMEM: Dulbecco′s Modified Eagle′s Medium.

**Figure S2. Morphological stability of micromolded alginate particles with or without encapsulated cells.** Human adipose stromal cells were loaded or not into alginate microparticles prepared in circular micromolds with a 150 µm diameter. The cells were suspended in a sterile 2% (w/v) alginate solution and charged into the molds by submitting the mold to centrifugation (300g for 2 minutes). After ionic cross-liking using an agarose gel loaded with 100 mM of CaCl_2_, alginate microparticles were stored in DMEM containing 1.8 mM CaCl_2_ at 37 °C. After 1, 7, 14, and 28 days, the microparticles were subjected to compression forces to 20% of deformation using a Microsquisher^®^. Height and diameter were measured, and Young’s modulus was calculated (n=6 for each condition). Results are expressed as means ± SEM. * represents a significant difference, p<0.05, two-way ANOVA, Tukey post-test


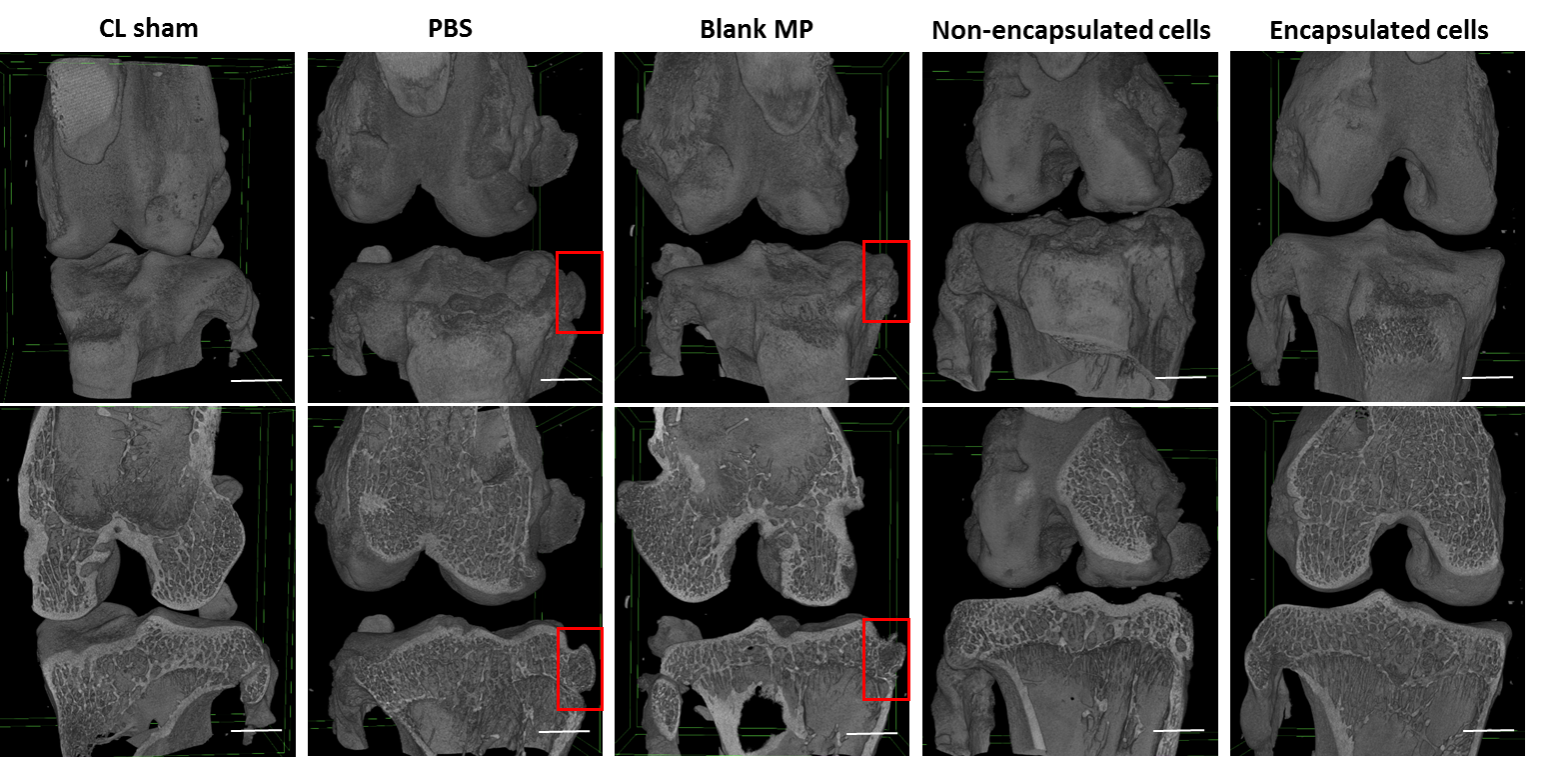


**Figure S3. Representative coronal micro-CT images of a rabbit knee joint.** Rabbits (n=6 per condition) underwent a destabilization of the right joint induced by ACLT. (A) Eight weeks after surgery, animals were randomly assigned into 4 groups. They were injected through a 26G needle with 200 µL of PBS, blank 2% (w/v) alginate microparticles (25 000 microparticles in 200 µL of culture medium, non-encapsulated human ASCs (500 000 cells in 200 µL of culture medium, or encapsulated human ASCs (500 000 cells in 25 000 2% (w/v) alginate microparticles in 200 µL of culture medium. Six weeks after the IA injection, the rabbits were euthanized. All operated (right), and non-operated (left, contralateral sham: CL sham) joints were dissected and used for image analysis. The experiment was performed with cells from one human donor (Donor B). Representative coronal micro-CT image of rabbit knee articulation (top: view from outside the joint, bottom: view from inside the joint) of all operated and non-operated joints at 14 weeks post-ACLT. Red frames indicate the presence of osteophytes. Scale bar 0.5 cm. Abbreviations: ACLT: anterior cruciate ligament transection, PBS: phosphate buffered saline, ASC: adipose-derived stromal cells, IA: intra-articular, MP: microparticles.

A

B

**Week 14**

**Week 20**

C

D

E

F

G

H

I

J

**Figure S4. Validation of OA in a rabbit model. ACLT causes significant changes in the tibial epiphysis bone architecture after 14 or 20 weeks.** Two independent animal experiments were performed to assess the time-course efficacy of encapsulated hASC. Rabbits (n=6 per condition) underwent a destabilization of the right joint induced by ACLT. Eight weeks after surgery, animals were randomly assigned into 4 conditions. They were injected through a 26G needle with 200 µL of PBS, blank 2% (w/v) alginate microparticles (25 000 microparticles in 200 µL of culture medium, non-encapsulated human ASCs (500 000 cells in 200 µL of culture medium, or encapsulated human ASCs (500 000 cells in 25 000 2% (w/v) alginate microparticles in 200 µL of culture medium. Six or twelve weeks after the IA injection, the rabbits were euthanized. All operated (right), and non-operated (left, contralateral sham: CL sham) joints were dissected and used for image analysis. The experiment was performed with cells from one human donor per animal experiment (Donors B and E). (A-D) Quantitative morphological assessment of the subchondral bone plate (SBP.Th, SBP.Po) of all operated and non-operated joints at 14 weeks (A and C) or 20 weeks (B and D) post-ACLT (E-J) Quantitative morphological assessment of the trabecular bone (Tb.Th, Tb.Sp, Tb.Po) of all operated and non-operated joints at 14 weeks (E, G and I) or 20 weeks (F, H and J) post-ACLT. * represents a significant difference, * p<0.05, ** p<0.01, *** p<0.001, Kruskal-Wallis test followed by Dunn’s comparison test, compared to CL sham. Abbreviations: OA: osteoarthritis, ACLT: anterior cruciate ligament transection, PBS: phosphate buffered saline, ASC: adipose-derived stromal cells, IA: intra-articular, MP: microparticles, SBP. Th: subchondral bone plate tissue, SBP. Po: subchondral bone plate porosity, Tb. Th: trabecular thickness, Tb. Sp: trabecular separation, Tb.Po: trabecular porosity.


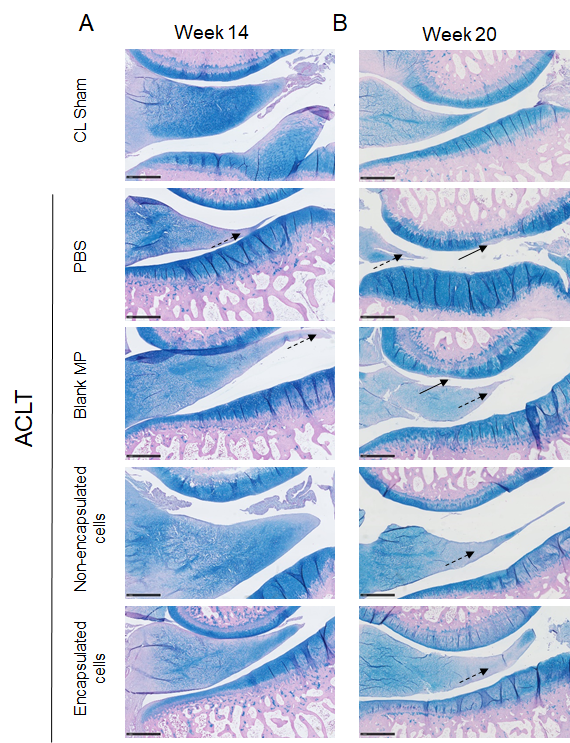


**Figure S5**. Alcian Blue staining of OA in ACLT rabbit model. Two independent animal experiments to assess the time-course efficacy of encapsulated hASC were performed. Rabbits (n=6 per condition) underwent a destabilization of the right joint induced by anterior cruciate ligament transection (ACLT). Eight weeks after surgery, animals were randomly assigned into 4 conditions and were injected through a 26G needle with 200 µL of PBS, blank 2% (w/v) alginate microparticles (25 000 microparticles in 200 µL of culture medium, non-encapsulated human ASCs (500 000 cells in 200 µL of culture medium, or encapsulated human ASCs (500 000 cells in 25 000 2% (w/v) alginate microparticles in 200 µL of culture medium. Six or twelve weeks after the IA injection, the rabbits were euthanized. All operated (right) and non-operated (left, contralateral sham: CL sham) joints were dissected and used for histological analysis. The experiment was performed with cells from one human donor per animal experiment (Donors B and E). Alcian Blue staining of all operated and non-operated joints at 14 weeks (A) or 20 weeks (B) post-ACLT. Scale bar 1 mm. Black arrows indicate slight discoloration and signs of loss of matrix glycosaminoglycan. Abbreviations: OA: osteoarthritis, ACLT: anterior cruciate ligament transection, PBS: phosphate buffered saline, ASC: adipose-derived stromal cells, IA: intra-articular, MP: microparticles.


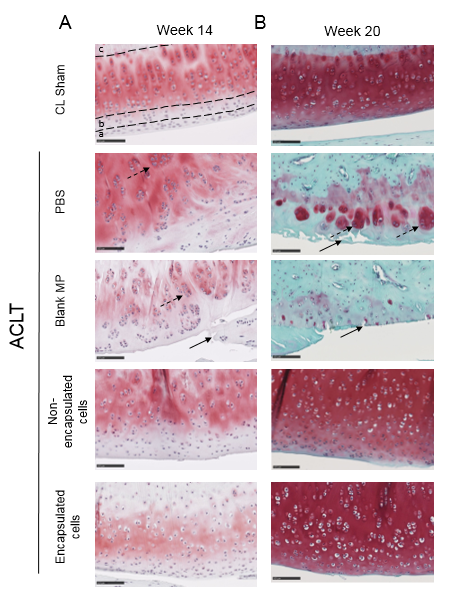


**Figure S6**. Histological analysis of rabbit knee after ACLT. Two independent animal experiments were performed to assess the time-course efficacy of encapsulated hASC. Rabbits (n=6 per condition) underwent a destabilization of the right joint induced by anterior cruciate ligament transection (ACLT). Eight weeks after surgery, animals were randomly assigned into 4 conditions. They were injected through a 26G needle with 200 µL of PBS, blank 2% (w/v) alginate microparticles (25 000 microparticles in 200 µL of culture medium, non-encapsulated human ASCs (500 000 cells in 200 µL of culture medium, or encapsulated human ASCs (500 000 cells in 25 000 2% (w/v) alginate microparticles in 200 µL of culture medium. Six or twelve weeks after IA injection, the rabbits were euthanized. All operated (right), and non-operated (left, contralateral sham: CL sham) joints were dissected and used for histological analysis. The experiment was performed with cells from one human donor per animal experiment (Donors B and E). Safranin-O staining of all operated and non-operated joints at 14 weeks (A) or 20 weeks (B) post-ACLT. Scale bar 100 µm. Black arrows indicate deep fibrillation or vertical fissures of the cartilage surface. Black dotted arrows indicate chondrocyte clusters in joint tissue. Various areas of non-calcified cartilage have been identified, (a) superficial zone; (b) median zone; (c) deep zone. Abbreviations: OA: osteoarthritis, ACLT: anterior cruciate ligament transection, PBS: phosphate buffered saline, ASC: adipose-derived stromal cells, IA: intra-articular, MP: microparticles.


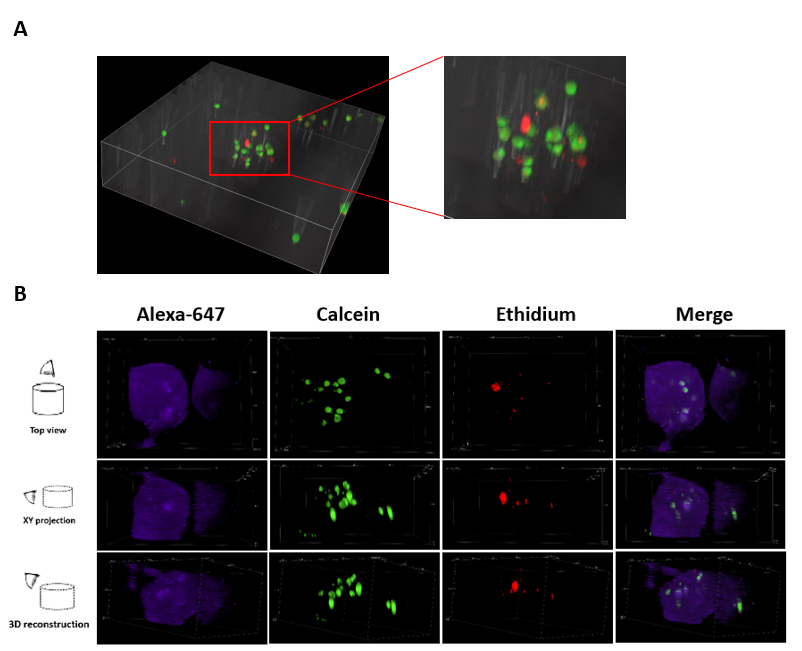


**Figure S7: Human mesenchymal stromal cells of adipose origin (hASC) encapsulated in an alginate hydrogel by micromolding.**

Encapsulation of hASCs by centrifugation and cross-linking of a 2% (w/v) alginate solution containing 3 million cells per mL of polymer in 150 µm diameter molds. (A) Confocal microscopy observation of live (green) and dead (red) cells after 2 months of encapsulation using calcein/ethidium labeling (voxel: 630 µm x 630 µm x 141 µm) with insert representing 1 particle containing 18 cells (magnification x 10). (B) Confocal microscopy observation of live (green) and dead (red) cells, using calcein/ethidium labeling after 10 days of encapsulation in 2% (w/v) alginate-Alexa-647, observed in far-red and shown in pseudocolor, ratio alginate-alexa-647/alginate = 0.1% (voxel: 329 µm x 400 µm x 206 µm).
